# Vitronectin Enrichment in Prostate Cancer Liver Metastases Promotes Adhesion and Survival

**DOI:** 10.1158/2767-9764.CRC-26-0305

**Published:** 2026-09-17

**Authors:** Jacob Egelberg, Rebecca Kim, Min J. Kim, Jeanne M. Dsouza, Alice Bernard-Tessier, Ramyar Molania, Varadha Balaji Venkadakrishnan, Jingjing Chen, Martin K. Bakht, Himisha Beltran

**Affiliations:** 1 Department of Medical Oncology, Dana-Farber Cancer Institute, Harvard Medical School, Boston, Massachusetts.; 2 Department of Pathology, Brigham and Women’s Hospital, Boston, Massachusetts.

## Abstract

The development of liver metastases in prostate cancer is associated with aggressive disease and poor prognosis. Because hepatocytes exhibit high metabolic activity with unique secretory profiles, we hypothesized that hepatocyte-to-tumor cell signaling plays a role in promoting liver metastasis. We evaluated single-cell transcriptomic data and metastatic tissue from patients with castration-resistant prostate cancer spanning androgen receptor (AR)–positive and AR-negative pathologies. Despite extensive intra- and intersample heterogeneity, communication analysis predicted vitronectin (VTN) engagement of tumor integrins as a common feature. VTN-positive hepatocytes were observed in prostate tumors with VTN accumulation in sinusoidal patches. Consistent with integrin activation, VTN treatment of AR-positive and AR-negative prostate cancer cells significantly promoted tumor cell adhesion, inhibited hypodiploid accumulation in accordance with survival effects, and stimulated FAK-dependent phosphorylation of ERK and AKT. FAK inhibition with defactinib abrogated VTN- and serum-mediated adhesion in a cell-specific manner and mitigated VTN-driven depletion of hypodiploid populations. These findings support a model in which dense intrasinusoidal VTN deposits capture metastatic prostate tumor cells in the liver and biochemically activate tumorigenic signaling, promoting tumor aggressiveness.

**Significance:**

Through the synthesis of single-cell transcriptomic data with experimental models, we identify VTN as a candidate microenvironmental mediator of prostate cancer liver colonization. VTN drives cell adhesion, inhibits hypodiploid accumulation consistent with cell survival, and activates oncogenic signaling, which may be inhibited by the dual FAK/PYK2 inhibitor defactinib.

## Introduction

Prostate cancer is the most common cancer and second leading cause of cancer death among men in the United States (1). Most prostate cancer–related deaths are due to the development of treatment resistance and progressive metastatic disease. Common initial sites of prostate cancer metastasis are bone and lymph nodes, with liver metastasis present in fewer than 5% of newly diagnosed patients with metastatic hormone-naive prostate cancer (2). However, up to 25% of patients with castration-resistant prostate cancer (CRPC) develop liver metastases over the course of their disease (3, 4). The presence of liver metastasis in either hormone-naive or castration-resistant prostate cancer is one of the strongest prognostic features, consistently associated with inferior outcomes across phase III clinical trials (4, 5).

Genomic analyses have reported enrichment of *MYC* amplification, *PTEN* deletion, and *PIK3CB* amplification in prostate cancer liver metastases compared with other metastatic sites (6). Patients with liver metastases also have higher circulating tumor DNA fractions with frequent and often co-occurring alterations involving *RB1*, *TP53*, and *PTEN* (7); loss of these tumor suppressors has been linked with aggressive disease, lineage plasticity, and androgen receptor (AR) independence (8–10). Patients with liver metastases have inferior responses to AR pathway inhibition (11, 12) as well as prostate-specific membrane antigen (PSMA)–targeted radioligand therapy (13), which might be, in part, due to lower or more heterogeneous AR and PSMA expression in liver metastases compared with other sites of disease (14).

Prostate cancer cells migrating to the liver can exhibit epigenetic remodeling consistent with microenvironment-mediated alterations (14). In other metastatic cancers, such as pancreatic cancer, it has been suggested that hepatic stellate and Kupffer cells communicate directly with metastatic tumor cells to promote growth (15). However, it remains unclear if metabolic features or intercellular signaling contributes to extravasation, neovascularization, or tumor progression in prostate cancer liver metastases. A better characterization of the biological drivers of prostate cancer liver metastasis could lead to new therapeutic strategies to improve patient outcomes.

Hepatocytes exhibit high metabolic activity with unique transcriptional and secretory profiles (16). Single-cell sequencing and enrichment analyses have identified specialized hepatic subpopulations with dedicated metabolic functions including gluconeogenesis, β-oxidation, lipid synthesis, and detoxification (17). Concordantly, liquid chromatography/tandem mass spectrometry of conditioned media from primary human hepatocytes has revealed a distinct hepatic secretome (i.e., hepatokines), consisting of nearly 700 proteins (18).

We hypothesized that soluble ligands secreted by resident hepatocytes promote prostate cancer tumorigenesis in the liver and contribute to poor patient outcomes. In this study, through integration of patient data with preclinical models, we identify vitronectin (VTN) as an important factor for promoting hepatic prostate cancer colonization. VTN enhances cell adhesion, inhibits hypodiploid accumulation consistent with cell survival, and activates the FAK, MAPK and PI3K pathways, which can be attenuated by treatment with the dual FAK/PYK2 inhibitor defactinib.

## Materials and Methods

### Cell culture

LNCaP (ATCC, RRID: CVCL_1379), C4-2 (ATCC, RRID: CVCL_4782), 22Rv1 (ATCC, RRID: CVCL_1045), 22Rv1-LMD (14), DU145 (ATCC, RRID: CVCL_0105), PC-3 (ATCC, RRID: CVCL_0035), and 22Rv1 PROX1 overexpression models (19) were cultured in RPMI-1640 medium without phenol red (Gibco #11-835-030) with 10% fetal bovine serum (FBS; Gibco #A5256701) and 1% penicillin/streptomycin (Gibco #15140-122). Media was replenished 3 times weekly. To passage, cells were washed once with phosphate-buffered saline (PBS; Corning #24-040-CV) and dissociated with TrypLE Express (Gibco #12-605-028) for 10 minutes at 37°C. Active enzyme was neutralized with an equal volume of complete media, and cells were spun at 270 × g for 5 minutes prior to resuspension in complete media. VCaP (ATCC, RRID: CVCL_2235) was cultured in DMEM basal media (Gibco #11965-092) with identical supplementation, NCI-H660 (ATCC, RRID: CVCL_1576) was cultured in RPMI-1640 supplemented with 2× insulin–transferrin–selenium (Gibco #41400045), 4 mmol/L GlutaMAX (Gibco #35050061), 5% FBS, 10 nmol/L hydrocortisone (Sigma #H0888), and 10 nmol/L β-estradiol (Sigma #E2758) in 6-well suspension culture plates (Greiner #657185), and PM154 and PM155 were cultured on collagen-coated (Gibco #A10483-01, 7 μg/cm^2^) plates in prostate-specific culture media media prepared as described previously without testosterone or Primocin (20). Media samples were tested for *Mycoplasma* contamination (InvivoGen #rep-mysv2-10) and confirmed to be *Mycoplasma*-free. Cells purchased from the ATCC were authenticated by the ATCC, routinely validated by marker expression and morphology, and passaged a maximum of 30 times. Models previously described by our lab were validated by marker expression and morphology and passaged a maximum of 50 times with no observed changes in growth kinetics or morphology.

Primary human hepatocytes (5-donor) were purchased from Thermo Fisher Scientific (Gibco #HMCPP5), thawed into hepatocyte thawing media (Invitrogen #CM7500), pelleted at 100 × g for 10 minutes at room temperature (the supernatant was poured off, without shaking or disturbing the pellet by aspiration), resuspended in 12 mL plating media (Invitrogen #CM3000, Invitrogen #A1217601), and plated in 6-well collagen-coated plates (5 μg/cm^2^; 2 mL and 1 × 10^6^ cells per well). After 24 hours, plating media was exchanged with 2 mL prewarmed maintenance media (Invitrogen #CM4000). Every 24 hours thereafter, conditioned maintenance media was collected and debris removed with a 300 × g spin for 5 minutes. The supernatant was aliquoted and frozen at −20°C, and fresh prewarmed maintenance media was added to the hepatocytes. Donor information about hepatocyte source patients is available in Supplementary Table S1.

### Ligand activation assays

Two days prior to sample harvesting, 5 × 10^5^ cells were plated in 6-well plates and allowed to adhere. One night prior to harvesting, complete media was removed and replaced with serum-free media. Inhibitors were added to serum-free media as required (trametinib: Selleck Chemicals #S2673; alpelisib: Selleck Chemicals #S2814; defactinib: Selleck Chemicals #S7654). On the day of harvesting, samples were incubated with 500 μL (enough to cover the plate surface) of 5 μg/mL VTN [recombinant VTN (rVTN): Abcam #ab217407; human plasma–purified VTN (hVTN): Sigma #CC080; mouse VTN: MedChemExpress #HY-P73484] at various timepoints prior to lysis with 100 μL protease (Roche #11836170001) and phosphatase (Thermo Fisher Scientific #78420) inhibitor–containing RIPA buffer (21). Lysis was allowed to proceed for 5 minutes at 4°C. Cells were collected in 1.5-mL Eppendorf tubes and pelleted at 14,000 × g for 10 minutes at 4°C. The supernatant was transferred to fresh 1.5-mL tubes, and the DC protein assay (Bio-Rad #5000111) was used to quantify total protein concentration. Samples of 30 μL and 50 μg protein were prepared from quantified lysate, complete RIPA buffer, and 4× Laemmli SDS-PAGE buffer (Bio-Rad #1610747). From the prepared samples, 20 μL (33.5 μg protein) was loaded on 4% to 15% precast protein gels (Bio-Rad #4561083) and run at 40 milliamps per gel until the desired endpoint.

### Immunoblotting

SDS-PAGE gels were transferred to 0.45-μm nitrocellulose membranes (Bio-Rad #1620115) at 100 V for 1 hour at 4°C. The membranes were blocked in 5% BSA for 1 hour at room temperature and probed with a primary antibody diluted in 1% BSA in Tris buffered saline with Tween (TBST) overnight at 4°C (Supplementary Table S2). The membranes were washed 3× in TBST for 3 minutes each before staining with a secondary antibody for 1 hour at room temperature. After staining, the membranes were washed 3× in TBST for 3 minutes each and submerged in Immobilon Classico Western HRP Substrate (Sigma #WBLUC0100) for 30 seconds immediately prior to imaging. When necessary, the membranes were stripped with Restore Western Blot Stripping Buffer (Thermo Fisher Scientific #21059) for 25 minutes at room temperature and reblocked prior to reprobing. Stripping was applied for the detection of total protein after blotting with phospho-specific antibodies.

### Adhesion assays and plate coating

Confluent cells were dissociated as described earlier (except NCI-H660) and washed once in 2 mL serum-free media. Cells were concentrated in 500 μL serum-free media, vortexed until homogeneous, counted, and allowed to rest at room temperature while experimental conditions were prepared. The concentrated stock of cells was then revortexed and rapidly diluted to 2 × 10^5^ cells per mL in 320 μL for each condition to test. One hundred microliters of cells was plated in 96-well plates in triplicate for each condition, and cells were allowed to attach for the specified amounts of time. After attachment, media was aspirated with care not to touch well bottoms, and wells were washed once with 100 μL PBS. Another 100 μL of PBS was added to the wells, and adherent cells were imaged by microscopy and quantified with CellTiter-Glo (Promega #G7570).

To coat plates with VTN (22), a 5 mg/mL stock was prepared by dissolving 100 μg VTN in 20 μL of distilled water (dH_2_O). Then, VTN was diluted to 5 μg/mL in PBS, and 100 μL was added to each well of a 96-well plate. The plates were placed in the incubator for 2 hours and then chilled overnight at 4°C. The plates were stored at 4°C until use, and PBS was aspirated prior to cell plating with care not to disturb the well bottom. Plates were washed once with PBS directly prior to use. For C4-2, 22Rv1, DU145, and PC-3 adherence on 6-well plates, VTN coating as sparse as 0.47 μg/cm^2^ was sufficient for spreading. For PM154 and PM155, coating with a density of 1.56 μg/cm^2^ was necessary to facilitate spreading.

To coat plates with polyHEMA (23), 1 g polyHEMA (Sigma #P3932) was dissolved in 100 mL 95% EtOH at 40°C overnight. Then, 40 μL or 1 mL was added to either 96-well plates or 6-well plates, respectively, and allowed to dry in a fume hood. Another round of polyHEMA was added and allowed to evaporate, and plates were stored at room temperature until use. Plates were washed twice with PBS directly prior to use.

### Immunohistochemistry

Formalin-fixed tissue was paraffin-embedded and sectioned. Slides were heated to 55°C for 15 minutes and immersed in xylene followed by serial immersion in reducing concentrations of ethanol for deparaffinization and rehydration. Then, slides were boiled in high-pH Antigen Unmasking Solution (Vector Labs #H-3301), peroxidase-inhibited with 3% H_2_O_2_ (Thermo Fisher Scientific #H325-500), and blocked with Background Punisher (Biocare Medical #BP974) for 4 minutes. The primary antibody was diluted into a blocking cocktail of secondary antibody host serum, PBS, and TBST and added to slides overnight at 4°C. Excess antibody was washed away, and staining was completed with the VECTASTAIN Elite ABC kit (Vector Labs #PK-6101). Hematoxylin (StatLab #HXHHEGAL) was applied for nuclear contrast.

### Immunofluorescence

Slides were heated, washed, unmasked, blocked, and probed with the primary antibody as described for immunohistochemistry (IHC). The next day, slides were washed with TBST and incubated with the secondary antibody. After secondary staining, ProLong NucBlue (Invitrogen #P36983) mounting media was applied and allowed to cure overnight at 4°C.

### Migration and chemotaxis assays

Migration assays were performed as previously described (24) with a minor modification to the establishment of cell-free zones on the culture plate. Rather than scratching a confluent monolayer, which introduces debris and varies in width, the gasket from a cryovial tube (CELLTREAT #229913) was applied to the center of 6-well plates. Light pressing creates a homogeneous watertight barrier between the plate interior and exterior. Then, 1.5 mL of cell-rich media was added to the edges of the plate and allowed to grow to confluence. At confluence, cells were serum-starved for 24 hours before the gasket was removed, allowing cells to migrate inward. For chemotaxis assays, 80 μL of either VTN diluted to 5 μg/mL in PBS or PBS alone was aliquoted into the center of the gasket and allowed to attach to the plate prior to cell seeding. Distance chemotaxed was quantified by calculating the Euclidean distance from a reference point to various points along the migrating cell front.

### Proliferation assays

Approximately 5,000 cells were plated in 96-well plates in complete media. After 24 hours, media was replaced with low-serum (1% FBS) media with or without conditions of interest. Media was replenished daily for 4 days before viable cells were quantified with CellTiter-Glo.

### Propidium iodide and apoptosis assays

For propidium iodide (PI) staining followed by flow cytometry, 8 × 10^5^ cells were seeded in 6-well plates under the specified conditions and allowed to attach. For C4-2, 22Rv1, PC-3, and DU145, cells were plated in 2 mL serum-free media with either 5 μg/mL VTN in dH_2_O or dH_2_O alone. For PM154 and PM155, 16.8 μg/mL VTN was used to facilitate spreading. After 24 hours, the supernatant was collected and cells were dissociated with TrypLE for 10 minutes. TrypLE was neutralized by dilution with supernatant, and cells were washed in 2 mL PBS before concentrating in 400 μL PBS. To fix cells, 800 μL 100% EtOH was added to each cell aliquot, and cells were allowed to sit overnight at 4°C. Prior to flow cytometry, cells and PBS were retrieved and allowed to equilibrate to room temperature. Cells were washed with 1 mL PBS and resuspended in 400 μL 1× PI with 1× RNase A in PBS (Abcam #ab139418). Staining was allowed to proceed for 30 minutes at 37°C before cells were filtered and placed on ice (Thermo Fisher Scientific #08-771-23). Flow cytometric analysis was performed immediately after filtering with the 561-nm yellow-green laser and 610/20 PE Texas Red filter.

Analysis of flow cytometry PI staining data was performed with flowCore V2.22.1 (25). Gating was performed by forward scatter area and side scatter area to remove debris and PE Texas Red-H and PE Texas Red-A to remove doublets (26). For cell-cycle classifications, Gaussian-kernel density estimation with the base density() R function was fit to PE Texas Red emission intensities for each cell line (pooling experimental conditions). First, the peak intensity was identified. Cells within 0.5 standard deviations (SD) of the peak were classified as G_1_ cells. Cells with intensity below the G_1_ peak minus 0.5 SDs were classified as hypodiploid. Then, twice the peak intensity was identified. Cells within 0.5 SDs of this value were classified as G_2_–M. Cells with intensities above the G_1_ peak plus 0.5 SDs and below the G_2_–M peak minus 0.5 SDs were classified as S. Finally, cells with intensities greater than 0.5 SDs above the G_2_–M peak were classified as >4N and excluded. Ranges used for cell-phase classification did not overlap.

For chemically induced apoptosis, 2 × 10^4^ cells were plated in 96-well plates in complete media. Cells were allowed to attach for 24 hours, serum-starved for another 24 hours, and then treated with an etoposide (Selleck Chemicals #S1225) concentration gradient in serum-free media with or without conditions of interest for 72 hours. Cells were washed once with PBS, and viable cells were quantified with CellTiter-Glo.

### Animal studies

The animal study was approved by the Dana-Farber Cancer Institute (DFCI) Institutional Animal Care and Use Committee (protocol number 18-020). Body condition scores less than or equal to 2 were used as the humane endpoint to terminate the study (27). Mice were monitored daily, and body weight was checked at least once a week. Reduction in body condition scores without reaching the study endpoint prompted checks at least twice a week. For orthotopic injection, cells were dissociated, counted, and portioned into aliquots of 2 × 10^5^ cells each. Aliquots were injected into the anterior prostate of 10-week-old male NOD/SCID gamma (NSG) mice (∼28 g; The Jackson Laboratory, RRID: IMSR_JAX:005557). Two mice per group were assigned to receive 50 μL intravenous injections of either 4 μg VTN diluted in PBS or PBS alone every other day for 54 days. All mice were euthanized on day 54 at the planned experimental endpoint; no mouse met the predefined humane endpoint before that time. Investigators were not blinded during experimentation, and no formal sample size calculation was performed, as all animal work presented is exploratory.

### Patient samples

All patient samples were handled according to the recognized ethical standards outlined by the Declaration of Helsinki. Autopsy tissue was acquired with informed consent from patients (institutional review board protocol numbers 20-000 and 19-883).

### Statistical analysis

Statistical analysis was performed in R (V4.4.2). For experimental data, the base t.test function was used for comparison of two sample measures of central tendency, and the base fisher.test function was used to quantify categorical associations. Fluorescent and bright-field images were analyzed using QuPath V0.7.0.

Single-cell analysis was performed with Seurat V5 (28), and statistical determinations were made with the FindMarkers or FindAllMarkers functions. Cells with few unique genes or total RNA per cell, or excessive RNA per cell, were excluded on a per-sample basis (29), and quality control parameters per sample are described in Supplementary Table S3. Then, samples were merged into a single Seurat object, log-normalized with NormalizeData, and variable genes identified with FindVariableFeatures. Normalized expression was scaled and centered with ScaleData, and principal components (*n* = 50) were constructed from variable genes with RunPCA. These components were used to construct a neighborhood graph (dims = 1:30) and cluster cells (Leiden resolution = 0.15) prior to dimensionality reduction via RunUMAP (dims = 1:30). To adjust for patient-associated batch effects, samples were integrated with Harmony prior Uniform Manifold Approximation and Projection (UMAP) generation (IntegrateLayers from SeuratWrappers V0.4.0, method = HarmonyIntegration; ref. 30).

CellChat V2.2.0.9001 was used for cell communication analysis (type = “truncatedMean”; trim = 0.1; raw.use = TRUE; ref. 31), fgsea V1.36.2 (bioRxiv 2021.02.01.060012) for gene set enrichment analyses (GSEA; seed = 123; ref. 32), and CopyKAT V1.1.0 for inferring copy-number profiles and aneuploidy (33). Unfiltered (DESeq2/cBioPortal) or FindAllMarkers-filtered (Seurat, default parameters) genes were ranked by log fold change (FC) prior to fgsea, and for analyses involving tumor cells, only cells belonging to tumor-annotated clusters with depleted immune signatures were included. For all *P* values, *, *P* ≤ 0.05; **, *P* ≤ 0.01; ***, *P* ≤ 0.001; ****, *P* ≤ 0.0001.

For bulk RNA sequencing (RNA-seq) analysis of GSE211448, DESeq2 V1.50.2 was used to identify differentially expressed genes between prostate and liver tumors. FCs were calculated from normalized counts and used as input to fgsea. For bulk RNA-seq analysis of Stand Up To Cancer (SU2C)–Prostate Cancer Foundation (PCF) transcriptomic data, polyA-selected fragments per kilobase million (FPKM) was read directly into R, filtered to samples from either prostate or liver tumors, and pseudocount-adjusted. Log FCs were calculated and input to fgsea.

Gene sets used in the study are provided in Supplementary Table S4, FindAllMarkers output is provided in Supplementary Table S5, and CellChat pathway and ligand–receptor outputs are provided in Supplementary Tables S6 and S7. In Supplementary Table S5, *P* values displayed as 0 reflect values below machine precision. In Supplementary Tables S6 and S7, *P* values displayed as 0 indicate values below the resolution of the CellChat permutation procedure (*n* = 100 permutations). These values do not represent exact probabilities of zero.

## Results

### Liver metastasis heterogeneity implicates intercellular signaling

**Figure 1.**
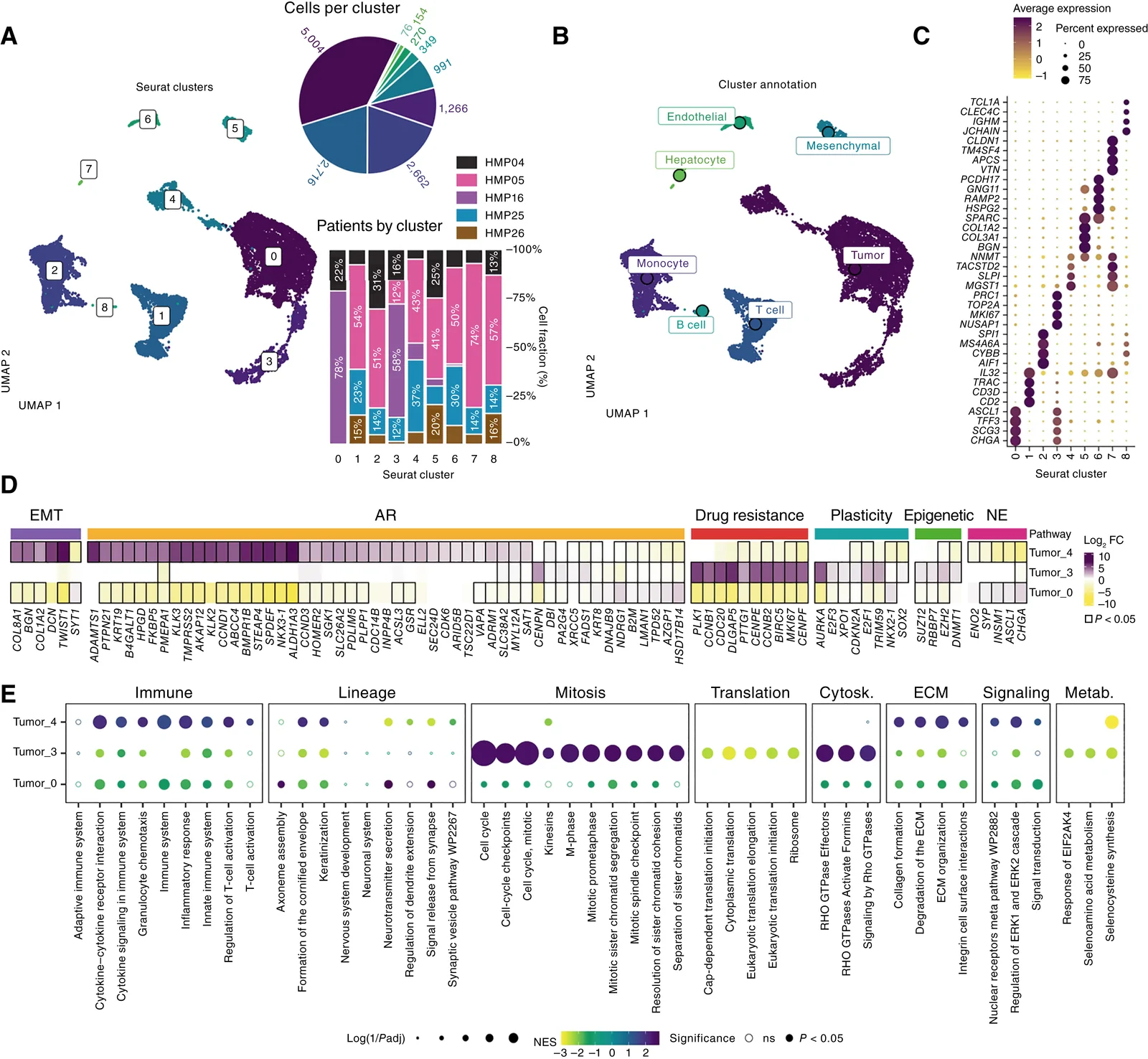
Liver metastases are highly heterogeneous with implications for tumorigenicity and cell signaling. **A,** Left, Harmony-integrated liver metastatic samples (*n* = 13,488 cells) from GSE210358 colored by Seurat cluster (resolution = 0.15). Each sample is derived from a unique patient; Right, pie and bar charts depicting the number of cells in each cluster and the fraction of cells belonging to each patient in each cluster, respectively. **B,** Cell-type cluster annotations determined by manual annotation, gene list enrichment analysis of top upregulated genes, and CopyKAT copy-number inference. **C,** Top marker genes for each cluster. Significance was determined with the FindAllMarkers function configured to apply a Bonferroni-corrected Wilcoxon rank-sum test. Significantly upregulated genes were ordered by adjusted *P* value, and the first four genes were selected for display. **D,** The parent Seurat object was subset to tumor cells (*n* = 6,226 cells), and differential expression analysis was conducted on every tumor cluster using the FindAllMarkers function. Resulting markers were subset to genes of interest and plotted. FindAllMarkers was configured to use a Bonferroni-corrected Wilcoxon rank-sum test for statistical determinations. **E,** Average log FCs of tumor marker genes were inputted to the fgsea function for GSEA, which uses a false discovery rate–adjusted permutation test to calculate significance. Cytosk., cytoskeleton; Metab., metabolism; NES, normalized enrichment score; ns, not statistically significant.

To characterize hepatocyte-to-tumor cell signaling in liver lesions, we evaluated single-cell transcriptomic data from five patients with prostate cancer liver metastases (Supplementary Table S3; refs. 34, 35). Dimensionality reduction after integration yielded a sample-mixed object with distinct transcriptomic clusters (Fig. 1A; Supplementary Fig. S1A–S1G). For identification of hepatocyte and tumor cell populations, we performed differential expression analysis between each cluster and all other clusters. Cell types were assigned according to cluster-level expression of established cell-type markers (36) and gene list enrichment analysis (37) of significantly upregulated genes (Fig. 1B; Supplementary Fig. S1H–S1J; Supplementary Tables S4 and S5). Because clusters 0, 3, and 4 were enriched with prostate cancer gene sets and cancer-associated genes such as *ASCL1*, *MKI67*, and *TACSTD2* (Fig. 1C) and were predominantly composed of cells with inferred aneuploidy, cells in these clusters that lacked expression of immune signature genes were defined as malignant (Supplementary Fig. S1K–S1N). In total, 154 hepatocytes in cluster 7 and 6,226 tumor cells in clusters 0, 3, and 4 were identified.

Expression of the neuroendocrine (NE) lineage markers *ASCL1* and *CHGA* in clusters 0 and 3, which minimally expressed *AR* and *NKX3-1*, suggested that dimensionality reduction captured NE prostate cancer (NEPC) pathology for malignant cells. Indeed, stratification of the integrated UMAP by histopathology confirmed that clusters 0 and 3 were comprised largely of cells from pathologically annotated NEPC samples, whereas cluster 4 consisted primarily of cells from CRPC-adenocarcinoma annotated samples (Supplementary Fig. S2). Seurat module scoring, which computes the difference in expression between genes in a given set and control genes from matched expression bins, generally aligned with pathologic annotations; clusters 0 and 3 showed enrichment of NE signature genes, whereas cluster 4 presented high adenocarcinoma scoring. However, conflicts between sample-level pathologic annotation and cell-level module scores arose for 24.1% of tumor cells and notable intrasample heterogeneity was evident, including cells that expressed neither adenocarcinoma nor NE markers (i.e., “double negative”). Because tumor clusters were associated with lineage markers but some cells did not express either CRPC or NEPC signature genes, we refer to clusters 0 and 3 as AR-depleted Tumor_0 and Tumor_3 and cluster 4 as AR-enriched Tumor_4.

The partitioning of malignant cells into transcriptionally distinct clusters could implicate hepatocyte-to-tumor cell communication if the expression of surface receptors or signaling genes differed between clusters. To elucidate the breadth of oncogenic cell states represented by the retrieved samples and how they might reflect divergent cell communication, we performed differential expression analysis between tumor clusters (Fig. 1D) and GSEA on resulting FCs (Fig. 1E).

Notably, AR-enriched Tumor_4 was associated with high expression of extracellular matrix (ECM) interaction pathway genes and upregulation of the epithelial–mesenchymal transition (EMT) transcription factor *TWIST1*, as well as markers including *BGN*, *COL1A2*, and *DCN*. This cluster also showed heightened expression of AR target genes *KLK2*, *KLK3*, and *NKX3-1* and the AR-linked surface receptors *BMPR1B*, *SLC26A2*, and *SLC38A2*. Conversely, AR-depleted Tumor_3 exhibited relative downregulation of AR targets, significant enrichment of cell-cycle pathways, and heightened expression of the mitotic checkpoint genes *PLK1*, *CENPE*, and *CENPF* and apoptosis resistance genes *BIRC5* and *PTTG1*. AR-depleted Tumor_0 showed stronger expression of NE markers and relative enrichment of the neurotransmitter secretion and signal release from synapse gene sets (Fig. 1D and E).

Taken together, prostate cancer liver metastases are highly heterogeneous with lesions comprised of pathologically distinct malignant cells defined by unique transcriptional profiles. This heterogeneity includes ECM components, secreted neuropeptides, and surface receptors, implicating intercellular communication.

### Ligand–receptor pairing predicts state-specific cell communication

Given that liver metastasis heterogeneity spans genes and pathways involved in cell–cell signaling, we applied CellChat to predict hepatocyte–tumor communication between hepatocytes and tumor clusters rather than hepatocytes and all tumor cells pooled together. All CellChat results reported here refer to pathways with significantly probable communication (*P* < 0.05), and the term CellChat probability refers to the relative strength of predicted signaling between pathways. Because clusters contain cells from multiple samples, inferred communication does not capture sample-specific signals or compare signaling events in one sample to the others.

**Figure 2.**
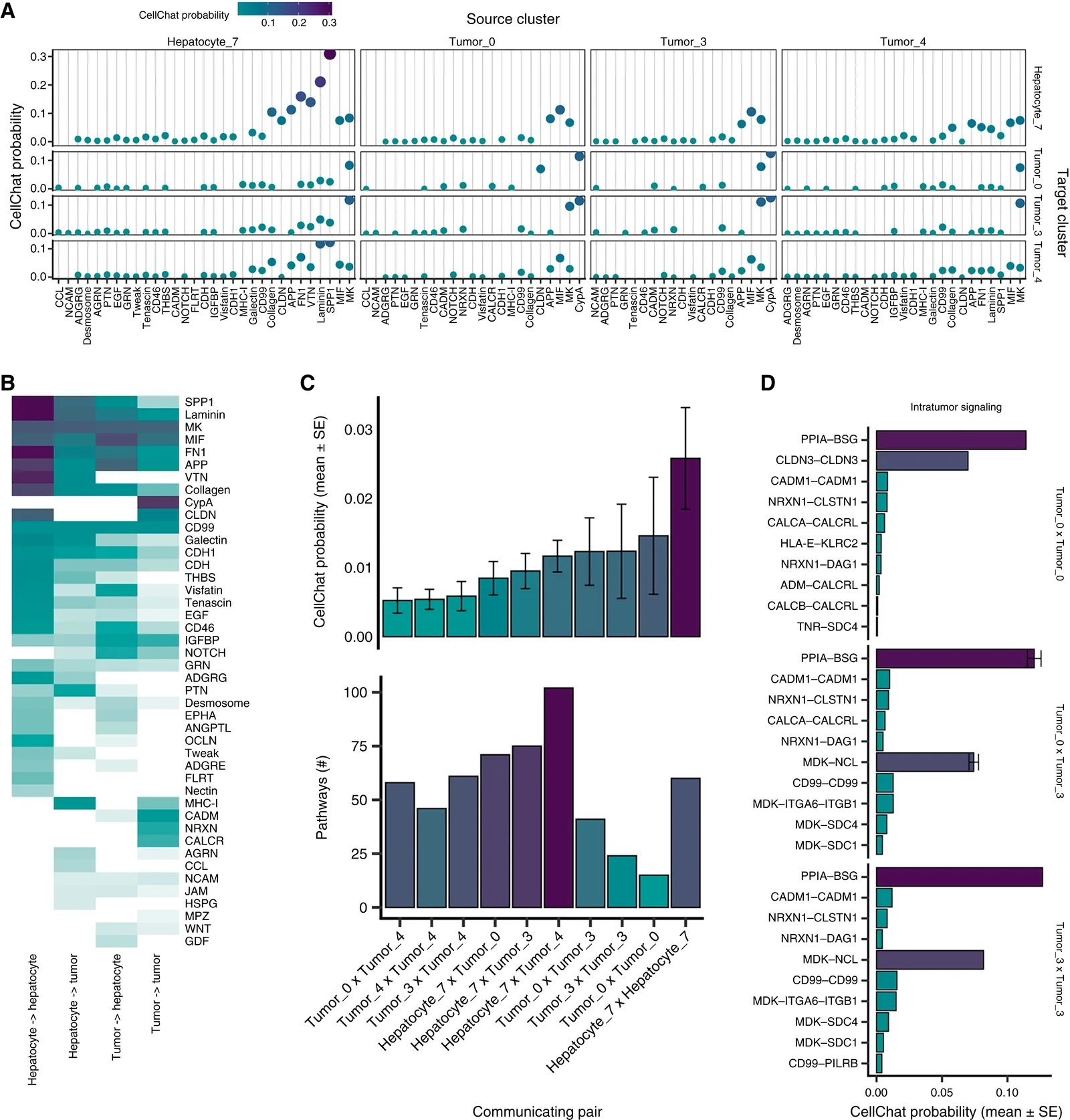
Tumor subtypes present distinct communication patterns. **A,** CellChat was used to infer intercellular communication from cluster ligand, receptor, and cofactor expression. CellChat uses a permutation test for statistical determinations, and all results depict significantly enriched pathways (*P* < 0.05). Plotted pathways were selected by averaging pathway-level CellChat probability across all groups and selecting pathways with means in the top 50th percentile. **B,** CellChat probability scores were averaged for each significantly enriched pathway across communication direction. For each direction, the top 30 pathways were selected. Due to overlap between pathways, 44 unique pathways are shown. **C,** CellChat probability scores were averaged or tallied for each communicating pair across significantly enriched pathways. All 10 predicted communicating pairs are shown. **D,** CellChat results were subset to communication between each pair (regardless of communication direction), and CellChat probability scores were averaged across communication direction for each interaction; scores for monodirectional interactions (i.e., autocrine signaling) have no associated standard error (SE) in this context. Interactions were ordered by mean probability, and the top 10 were selected for plotting.

Overall, hepatocytes were predicted to be involved in more communication than any tumor cluster (ligand expressing: 70 pathways in hepatocytes, 42 in Tumor_0, 42 in Tumor_3, and 56 in Tumor_4; receptor expressing: 68 pathways in hepatocytes, 56 in Tumor_0, 58 in Tumor_3, and 63 in Tumor_4). Much of this estimated communication involved autocrine or paracrine signaling among hepatocytes, as the highest CellChat probability values were observed for hepatocyte-to-hepatocyte SPP1 and laminin signaling (Fig. 2A and B; Supplementary Tables S6 and S7). Strong tumor-to-tumor signaling was inferred for the CypA pathway, whereas the strongest predicted hepatocyte-to-tumor signaling involved ECM components such as SPP1, laminin, and fibronectin. However, non-ECM midkine (MK) signaling also showed strong probability scores for hepatocyte-to-tumor, tumor-to-tumor, and hepatocyte-to-hepatocyte communication.

As expected, notable heterogeneity was observed between tumor clusters. Tumor_3 and Tumor_4 but not Tumor_0 were estimated to receive APP communication. Although both AR-depleted clusters were strongly predicted to receive CypA signaling, AR-enriched cluster Tumor_4 was not. Conversely, Tumor_4 was strongly predicted to receive migration inhibitory factor (MIF) signals from tumor cells and hepatocytes, but activation of this pathway was not inferred for either Tumor_3 or Tumor_0. Interestingly, although both Tumor_0 and Tumor_3 were predicted to send Notch signals, only Tumor_4 was predicted to receive them.

Calculating average CellChat probability for all communication pairs further supported hepatocyte-to-hepatocyte communication as relatively stronger compared with other inferred communication (Fig. 2C). This also revealed autocrine or paracrine signaling among Tumor_0 and Tumor_3 cells to be particularly strong. After these communicating pairs, hepatocyte-to-tumor communication for each tumor cluster showed strong CellChat probability. Examination of intratumor estimated signaling pointed to PPIA–BSG interactions, a component of CypA signaling, as enriched, as well as nucleolin-mediated MK signaling (Fig. 2D). Only Tumor_0 was predicted to form CLDN3-involved tight junctions.

In aggregate, hepatocytes seemed more involved in cell communication compared with tumor cells alone, but inter- and intracluster tumor signaling remained notable and varied with oncogenic state.

### CellChat predicts pan-cluster VTN communication

**Figure 3.**
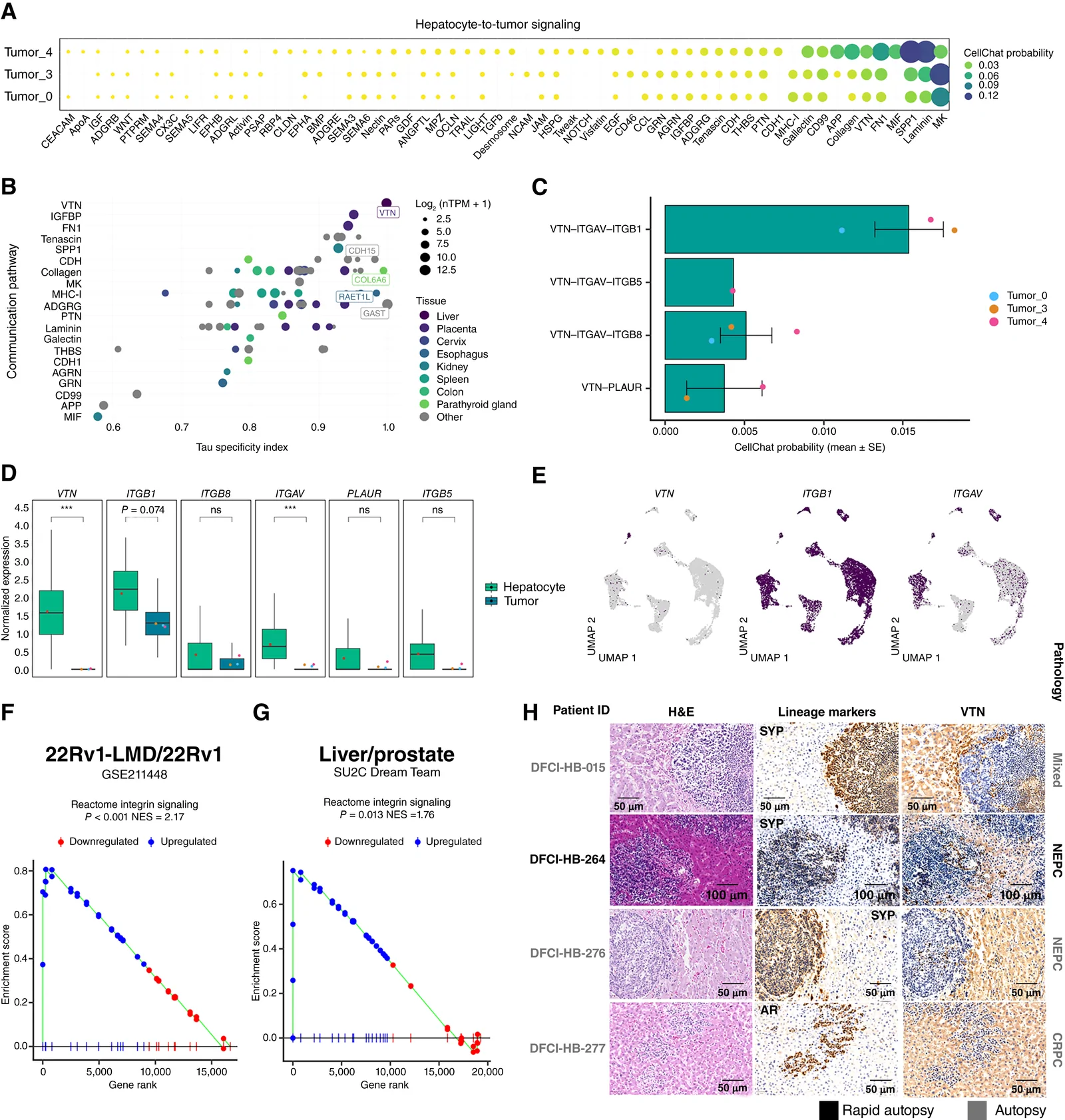
Tumor-directed VTN signaling is a shared across clusters. **A,** CellChat results were subset to communication predicted to originate from hepatocyte-annotated cluster 7 and be received by tumor-annotated clusters 0, 3, and 4. Mean CellChat probabilities were calculated for each pathway across tumor clusters, pathways were ordered by their mean value, and the top 60 were selected for plotting. **B,** Ligands for the top 20 pathways in (**A**) were retrieved from CellChat results and annotated with tissue-level expression from The Human Protein Atlas bulk RNA-seq data. Tissue specificity was calculated for each ligand with the Tau index, and pathways were ordered by the mean Tau index of their ligands. **C,** CellChat-predicted ligand–receptor pairs involved in VTN signaling were retrieved from the CellChat object. CellChat probability scores and *P* values (permutation test) were retrieved for each pair and communicating cluster and subset to significant (*P* < 0.05) hepatocyte-to-tumor communication. Ligand–receptor pairs were ordered by their median CellChat probability score across tumor clusters for plotting. **D,** Single-cell normalized expression data were extracted for CellChat-annotated ligand–receptor pairs involved in VTN signaling. Boxplots show the distribution of cell-level expression for hepatocytes and tumor cells, and points show cluster means. Significance was calculated with DESeq2 from sample and tumor/hepatocyte-level pseudobulked counts. ns, not statistically significant. **E,** UMAPs colored by binary *VTN*, *ITGB1*, or *ITGAV* expression. **F** and **G,** Count and FPKM matrices were obtained from GSE211448 and cBioPortal for the 22Rv1 orthotopic model and SU2C Dream Team datasets, respectively. For 22Rv1, DESeq2 was applied to normalize counts and calculate FCs between 22Rv1-LMD and primary 22Rv1. For SU2C Dream Team data, FCs were calculated from pseudocount-adjusted FPKMs. Then, FCs were inputted to fgsea for GSEA, which uses a false discovery rate–adjusted permutation test to calculate significance. Enrichment results for Reactome integrin signaling (R-HSA-354192) are shown. **H,** Liver tissue was harvested at autopsy (Supplementary Table S8) and formalin-fixed, paraffin-embedded, and sectioned. Slides were stained with hematoxylin and eosin (H&E), relevant lineage markers, and VTN. ***, *P* < 0.001.

To identify hepatocyte-driven oncogenic signaling that might contribute to the poor outcomes seen for patients with liver metastases, we filtered predicted intercellular interactions to those with strong outgoing CellChat probability for hepatocytes and strong incoming CellChat probability for tumor cells. MK signaling yielded the highest CellChat probability and was predicted to occur in all three tumor clusters (Fig. 3A). In contrast, other estimated hepatocyte-to-tumor communication signals were target specific, with no ADGRL signaling predicted in Tumor_4 and no SEMA5 signaling predicted in Tumor_3. Despite inferred BMP signaling from hepatocytes to Tumor_4 and Tumor_3, BMP was not predicted to be received by Tumor_0.

Then, to ensure the specificity of predicted hepatocyte-to-tumor communication for the liver microenvironment, we calculated the Tau specificity index for all pathway ligands from The Human Protein Atlas tissue expression data (38, 39). VTN, the primary ligand for VTN signaling, was identified as the most liver-specific ligand (Fig. 3B), and VTN signaling was inferred in all three tumor clusters (Fig. 3A). Integrin αV was strongly predicted to receive VTN signals by heterodimerizing with integrins β1, β5, or β8 (Fig. 3C) and urokinase plasminogen activator receptor (uPAR; gene *PLAUR*) was also predicted to bind VTN to a lesser extent. The most favored binding partner of integrin αV was integrin β1, with heterodimerization and VTN binding predicted in all tumor clusters.

We verified the robustness of inferred VTN communication by validating that ligand and receptor expression matched the direction of predicted signaling. As expected, *VTN* was significantly upregulated in hepatocytes, whereas integrins were expressed sporadically by all tumor cells (Fig. 3D and E). In an external dataset of single-cell transcriptomics aggregated by The Human Protein Atlas (40), the highest *VTN* expression was observed in hepatocytes, whereas prostate cells demonstrated negligible expression [hepatocyte counts per million (CPM) = 2,942; prostatic club, hillock, and glandular cell CPM = 0.1; basal prostate cell CPM = 0; Supplementary Fig. S3A]. Similarly, DepMap expression and proteomic data (41) confirmed that prostate cell lines express notably more mRNA and protein for VTN receptors than VTN itself (Supplementary Fig. S3B–S3P).

Sensitivity, downsampling, and generalizability analyses, including examination of two bulk transcriptomic prostate cancer datasets, and IHC staining further substantiated that the observed patterns of ligand and receptor expression could plausibly produce VTN communication. VTN signaling remained robust to modulation of truncated mean trim and partially robust to other CellChat expression aggregation metrics; communication was predicted using the thresholdedMean and triMean but not the median summary statistics (Supplementary Fig. S4A and S4B). Predicted signaling also remained after randomly downsampling tumor clusters to equal the quantity of hepatocytes (Supplementary Fig. S4C and S4D) and after merging tumor cells with an independent dataset of 3,262 hepatocytes (Supplementary Fig. S4E–S4I; ref. 17). In bulk RNA-seq data, GSEA of both datasets identified significant enrichment of Reactome integrin signaling in liver metastatic prostate tumors (Fig. 3F and G). Experimentally, VTN expression was detectable at the protein level in liver metastases from patients with NEPC and CRPC taken at the time of autopsy (Supplementary Table S8), with hepatocyte-specific VTN staining and extracellular VTN puncta seen within prostate tumors (Fig. 3H).

These results pointed to VTN signaling as a recurrent and enriched feature of prostate cancer liver metastases.

### VTN promotes prostate cancer cell adhesion

**Figure 4.**
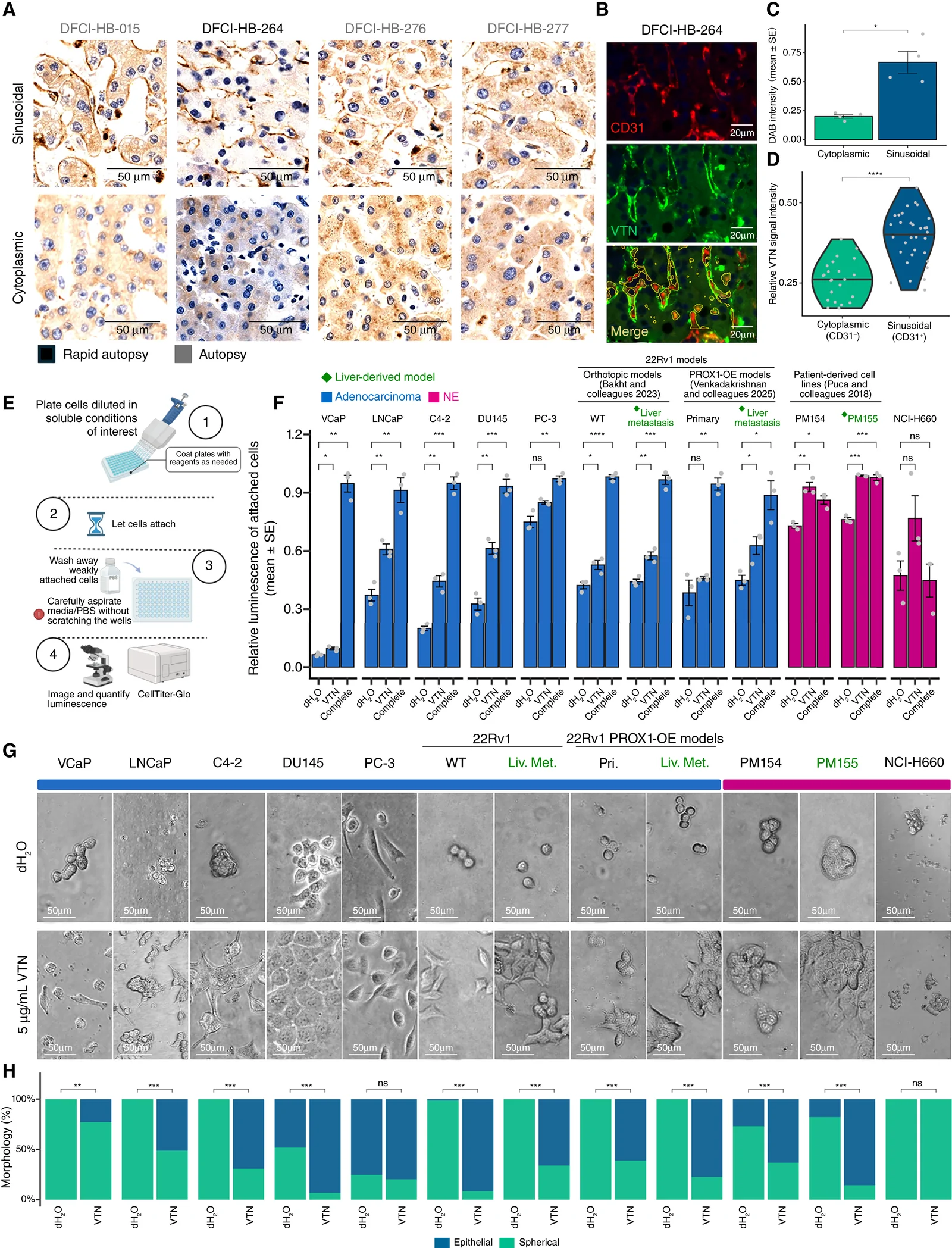
VTN localizes to the hepatic sinusoid lumen and promotes prostate cancer adhesion and spreading. **A,** Representative sinusoidal or cytoplasmic VTN staining from autopsy-harvested tumor tissue. **B,** Rapid autopsy tissue from DFCI-HB-264 was immunostained for CD31 (red) and VTN (green). Fluorescent images were loaded into QuPath, and CD31^+^ regions were defined with the native pixel classification thresholder algorithm. Individual CD31/red and VTN/green channels are shown, as well as a merged image in which CD31^+^ regions are outlined in yellow. **C,** From IHC staining, 20 representative cytoplasmic and sinusoidal regions per patient were marked with points. Point-level 3,3′-diaminobenzidine (DAB) intensity was averaged within each patient and staining pattern. Bars depict four biological replicates (from the four patients), and statistical indications show the results of a paired two-tailed *t* test. **D,** From immunofluorescence staining of DFCI-HB-264 liver tissue, within CD31^+^ regions and across the whole image, VTN+ areas were defined with the QuPath pixel classification thresholder algorithm retaining parent annotations (if a VTN+ region is within a CD31^+^ region). VTN intensities were normalized to the maximum value, regions were filtered to those with areas greater than 30 μm^2^, and values were compared between CD31^+^ (sinusoidal) and CD31^−^ (cytoplasmic) regions. Each point corresponds to one within-section region (*n* = 23 cytoplasmic; *n* = 32 sinusoidal), and statistical determinations were made with a two-tailed Welch *t* test. **E,** Schematic outlining an assay for cell adhesion with soluble or adherent independent variables. **F,** The described adhesion assay was performed on cells in serum-free media with or without 5 μg/mL VTN and allowed to adhere for 24 hours (48 hours for VCaP). For all cell lines except PM154 and PM155: Complete, use of complete media as a positive control; for PM154 and PM155, complete, plating on collagen-coated plates. Bars show three biological replicates, and significance was calculated with two-tailed Welch *t* tests. ns, not statistically significant; OE, overexpression; WT, wild-type. **G,** Representative images depicting VTN-associated morphologic transitions after plating in serum-free media. Liv. Met., liver metastatic 22Rv1 from orthotopic or PROX1-expressing models; Pri., 22Rv1 PROX1-expressing cells. **H,** Cells of epithelial and spherical morphology from representative images of attached VTN or control-treated cells after plating with serum-free media were counted and compared. Statistical determinations were made from individual images taken from the same experiment with a two-tailed Fisher exact test. [**E,** Created in BioRender. Egelberg, J. (2026) https://BioRender.com/5blud6q.] *, *P* ≤ 0.05; **, *P* ≤ 0.01; ***, *P* ≤ 0.001; ****, *P* ≤ 0.0001.

Supporting a potentially protumorigenic function, VTN is well-characterized as a cell adhesion factor (42) and deeper examination of histologic VTN staining showed localization to the luminal lining of hepatic sinusoids (Fig. 4A), the predominant site of tumor cell arrest during metastasis (43). Sinusoidal localization was confirmed by co-immunofluorescence of VTN with platelet and endothelial cell adhesion molecule 1 (CD31), an endothelial transmembrane receptor (Fig. 4B). Quantification of VTN intensity by IHC across patients and a within-section region of interest–level comparison of immunofluorescent staining (DFCI-HB-264 tissue) demonstrated significantly higher intensity of signal in sinusoid lumina compared with hepatocyte cytoplasm (Fig. 4C and D).

To validate inferred VTN-to-tumor communication and possible tumorigenic effects, we developed a high-throughput cell adhesion assay (Fig. 4E, see “Materials and Methods”) and screened adenocarcinoma and NEPC cell lines for attachment following VTN exposure. VTN significantly promoted adhesion in multiple AR-positive and NEPC/AR-negative models, including VCaP, LNCaP, C4-2, PM154, PM155, and DU145 (Fig. 4F), demonstrating a broad relevance of VTN responsiveness. PC-3, subcutaneously implanted/primary PROX1-expressing 22Rv1, and NCI-H660 showed a nonsignificant trend, highlighting model-specific heterogeneity. In all cell lines except PC-3 and NCI-H660, VTN was sufficient for cell spreading with increased cell fractions, assuming an epithelial morphology as opposed to the spherical form typical of suspended cells (Fig. 4G and H).

Screened cell lines included three liver metastatic models previously established by our lab: liver metastatic 22Rv1 (22Rv1-LMD), derived from liver-metastasized 22Rv1 following prostate orthotopic injection (14); PM155, developed by long-term culture of liver metastasis tissue from a patient with NEPC (20); and liver metastatic PROX1-expressing 22Rv1, isolated from liver metastasis following PROX1 overexpression in 22Rv1 and subcutaneous injection (19). Comparison of liver metastatic model adhesion with their most similar pair-matched controls (22Rv1 for 22Rv1-LMD, bone metastasis–derived NEPC model PM154 for PM155, and primary PROX1-expressing 22Rv1 for liver metastatic PROX1-expressing 22Rv1) revealed universally heightened VTN-mediated attachment among liver metastasis models (Supplementary Fig. S5). Although CellTiter-Glo luminescence–measured effect sizes were nonsignificant for 22Rv1 versus 22Rv1-LMD and PM154 versus PM155, liver metastasis of PROX1-expressing 22Rv1 was associated with significantly increased signal. Despite nonsignificant luminescent signals, the observed trend of all models tested aligned with manual cell counts from images of well centers, which showed significant differences (Supplementary Fig. S5).

These findings extend VTN’s canonical role as a cell adhesion and spreading factor to prostate cancer cells and provide a functional link to disease progression.

### Exposure to VTN inhibits hypodiploid accumulation and activates MAPK and PI3K

**Figure 5.**
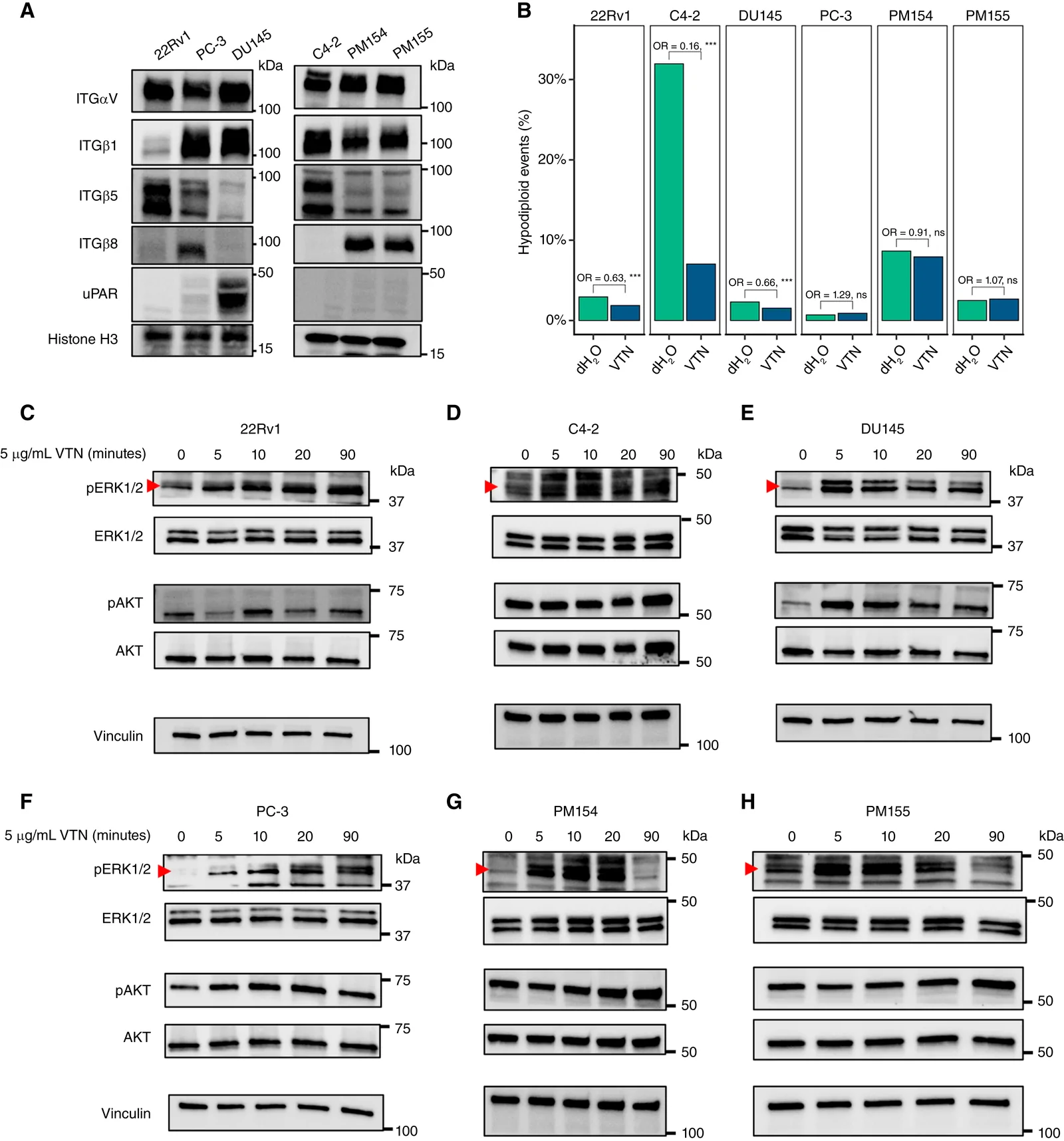
VTN promotes cell survival and stimulates MAPK and PI3K. **A,** Cells undergoing active culture were aliquoted, pelleted, and lysed, and immunoblotting was performed as specified in “Materials and Methods.” ITG, integrin. **B,** Cells were plated in 6-well plates in 2 mL serum-free media with either 5 μg/mL VTN in dH_2_O (for C4-2, 22Rv1, PC-3, and DU145), 16.8 μg/mL VTN in dH_2_O (for PM154 and PM155, necessary to facilitate spreading), or dH_2_O alone and allowed to adhere for 24 hours. Cells were harvested for PI staining followed by flow cytometry, and data were gated to exclude debris and doublets. Hypodiploid cell fractions were calculated as described in “Materials and Methods,” and statistical significance was determined from individual representative experiments with a two-tailed Fisher exact test. ns, not statistically significant. **C–H,** Cells were plated on 6-well plates, serum-starved overnight, and exposed to VTN for the specified amounts of time. PM154 and PM155 were stimulated with the same concentration of VTN as the other cell lines. After exposure, cells were lysed, and immunoblotting was performed as specified in “Materials and Methods.” Red triangles indicate the pERK bands. ***, *P* < 0.001.

CellChat predicted that VTN binds tumor cell integrins, and cell adhesion and spreading are predominantly facilitated by integrin activation (44). To investigate potential VTN–integrin binding, we verified protein-level expression of CellChat-predicted VTN receptors in a subset of models by immunoblotting. Consistent with CellChat’s identification of integrin αVβ1 as the most likely VTN binding partner, all cells evaluated were positive for integrin αV and integrin β1 protein (Fig. 5A). CellChat had also identified AR-positive Tumor_4 as the only cluster to bind VTN with integrin αVβ5. In agreement with this prediction, Western blotting showed strong expression of integrin β5 in 22Rv1 and C4-2, with relatively weak or non-expression in the AR-negative cell lines PC-3, DU145, PM154, and PM155. These results confirmed the feasibility of VTN–integrin binding and suggested that VTN could modulate integrin-downstream phenotypes.

To this end, previous work has highlighted the role of integrin signaling in apoptosis inhibition, and VTN has been linked to anoikis resistance in mesenchymal stem cells (45). Therefore, we assayed cells for hypodiploid accumulation within 24 hours of VTN stimulation with PI staining followed by flow cytometry. Apoptotic cells undergo rapid DNA cleavage, resulting in weak PI signal and increased hypodiploid events (46). Results indicated that VTN significantly inhibited the frequency of hypodiploid events in 22Rv1, C4-2, and DU145, with a nonsignificant but similar trend in PM154 (Fig. 5B; Supplementary Fig. S6). Neither PC-3 nor PM155 responded significantly to VTN in this context.

We also screened cells for VTN-mediated integrin-downstream signaling, including phosphorylation of ERK, AKT, YAP, and β-catenin, and NOTCH activity (47). VTN stimulation increased phosphorylation of ERK in all tested cell lines, as well as AKT in DU145 and PC-3 (Fig. 5C–H). Five-minute induction of VTN in 22Rv1 reduced pAKT, which may reflect intact PTEN-mediated negative feedback (48) and is consistent with its basal PI3K activity indicated by AKT phosphorylation (Supplementary Fig. S7). No consistent changes were seen in YAP or β-catenin phosphorylation or NOTCH activity (Supplementary Fig. S8).

Beyond adhesion, these findings suggest that VTN promotes the survival of, and activates oncogenic linked signaling in, adenocarcinoma and NEPC cell lines, likely as a result of integrin engagement.

### VTN-induced effects are robust to technical variability

However, because VTN was associated with reduced hypodiploid fractions, we revisited adhesion assay luminescent signals to validate that results predominantly reflect attachment and not VTN-mediated survival. 22Rv1, DU145, and PC-3 were chosen for validation assays because of the concordance observed between their adhesion and PI results; in 22Rv1 and DU145, VTN-induced intensity shifts allow for CellTiter-Glo luminescence to reflect survival rather than adhesion, whereas PC-3 responded nonsignificantly in both adhesion and PI assays and provided a control.

**Figure 6.**
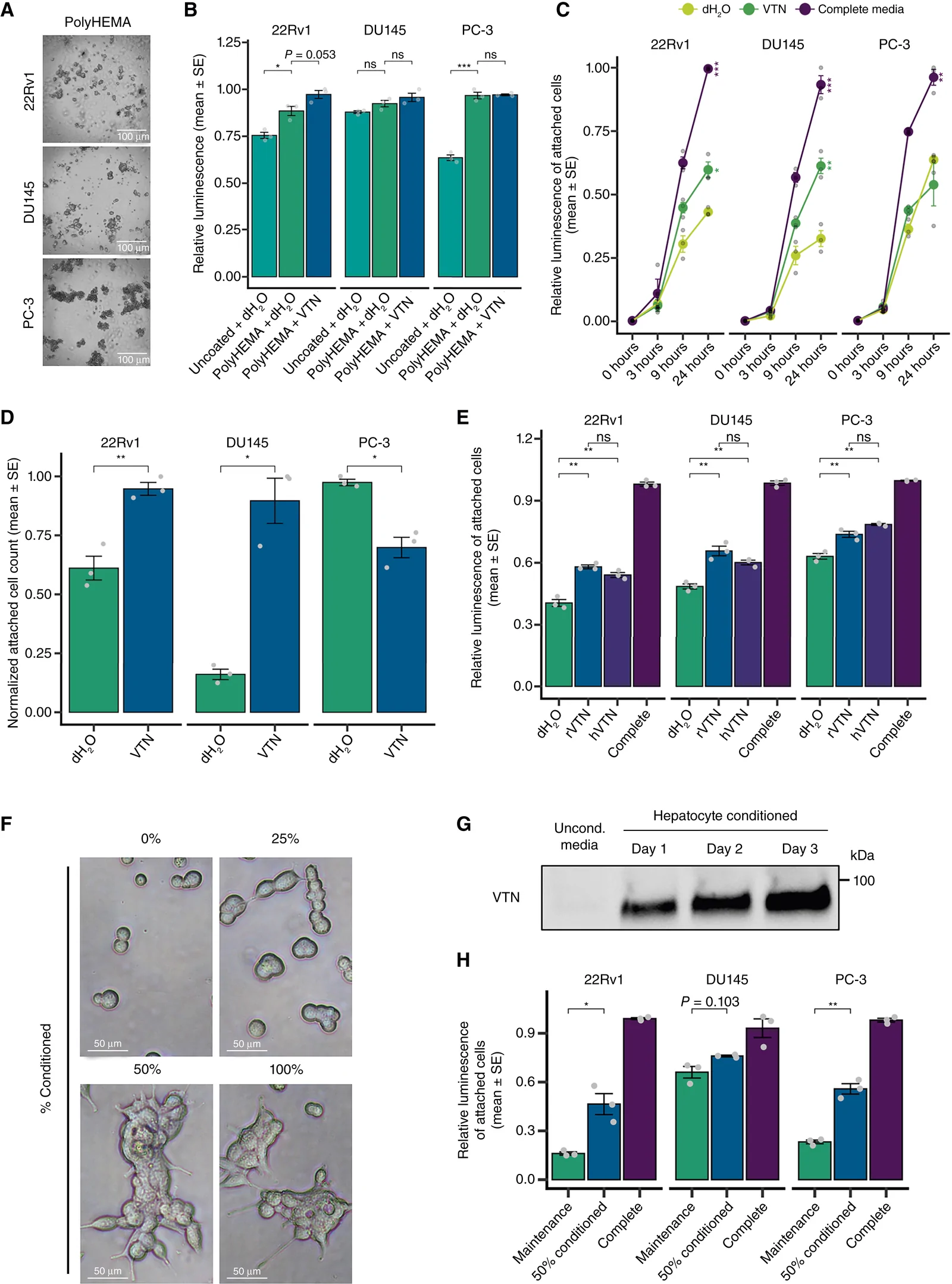
The adhesion effects of VTN are robust across assay conditions and biological sources. **A,** Representative microscopy of prostate cancer cells plated on polyHEMA-coated plates depicting the abrogation of cell attachment and successful spheroid formation. **B,** Adhesion assays were performed with polyHEMA-coated plates and serum-free media with or without 5 μg/mL VTN. Bars show three biological replicates, and significance was calculated with two-tailed Welch *t* tests. ns, not statistically significant. **C,** Cells were subjected to a time-course adhesion assay in serum-free media with or without 5 μg/mL VTN. Bars show three biological replicates, and significance was calculated for the 24-hour timepoint with two-tailed Welch *t* tests. **D,** Images were taken of well centers from the 24-hour timepoint, and cells within captured fields of view were counted. Results are for three biological replicates, and two-tailed Welch *t* tests were used for statistical determinations. **E,** Adhesion assays were performed in serum-free media with 5 μg/mL rVTN, 5 μg/mL human plasma–purified VTN (hVTN), or no VTN. Complete, use of complete media as a positive control. Bars show three biological replicates, and significance was calculated with two-tailed Welch *t* tests. **F,** Conditioned media was recovered from 5-donor human plateable hepatocytes (first collection period/day 1). An adhesion assay was performed with 22Rv1 and 100% conditioned media serially diluted 1:1 for seven timepoints. Images show representative regions of adhered or suspended 22Rv1 cells following exposure to 100%, 50%, 25%, or 0% conditioned media. **G,** Conditioned media (24 μL) was directly mixed with 8 μL of 4× SDS-PAGE and immunoblotted for VTN as specified in “Materials and Methods.” As media was collected and replenished every 24 hours, lane labels indicate the period of media collection since hepatocyte plating (day 1 corresponds to collection on day 1 since plating). Uncond. media, unconditioned hepatocyte maintenance media. **H,** An adhesion assay was performed with basal hepatocyte maintenance media, 50% hepatocyte-conditioned maintenance media, or complete culture media as a positive control (RPMI-1640 with 10% FBS). Complete, use of complete culture media as a positive control; maintenance, use of maintenance media as the basal serum-free media (rather than RPMI-1640). Bars show three biological replicates, and significance was calculated with two-tailed Welch *t* tests. *, *P* ≤ 0.05; **, *P* ≤ 0.01; ***, *P* ≤ 0.001.

Adhesion assays were repeated with polyHEMA-coated plates that prevent cell attachment (23). We reasoned that, if VTN-mediated survival skews CellTiter-Glo readouts, plating on polyHEMA would yield a reduction in CellTiter-Glo luminescence and VTN treatment would abolish this effect. However, we observed that plating on polyHEMA significantly increased luminescent signals for 22Rv1 and PC-3 relative to normal plates, with a nonsignificant but similar trend observed for DU145 (Fig. 6A and B). We also performed a time-course experiment to ensure that measured signals matched the temporal nature of cell adherence and, at the 24-hour timepoint, manually counted adhered cells from representative microscopy prior to CellTiter-Glo quantification. Luminescent intensity increased over time and manual counts closely mirrored luminescent readings (Fig. 6C and D). Consistent with high interassay variability, PC-3 showed fewer attached cells following VTN exposure. Nonetheless, this was captured by both CellTiter-Glo signal and microscopy, supporting their concordance.

These results suggest that VTN-mediated alteration of hypodiploid fractions does not meaningfully skew bulk CellTiter-Glo readouts toward lower values and reflect PI staining showing that only a fraction of suspended cells exhibit hypodiploid staining intensity regardless of VTN exposure. Rather, they indicate that CellTiter-Glo readouts underestimate the true fraction of adhered cells, as the process of adherence associates with reduced luminescence.

To further establish the robustness of VTN-induced effects and better model tumor–VTN interactions *in vivo*, we exposed cells to a continuous surface spotted with VTN foci. Within 24 hours of plating with serum-free media, cells which were VTN reactive in the adhesion assay flattened and spread exclusively in VTN-coated regions (Supplementary Fig. S9). We also assayed cells for stimulation with murine VTN, to ensure that earlier gene set enrichment results (Fig. 3F and G) plausibly reflect VTN-mediated changes (Supplementary Fig. S10A); human-purified VTN (Fig. 6E; Supplementary Fig. S10B), which would retain native posttranslational modifications; and hepatocyte-conditioned media (Fig. 6F–H; Supplementary Fig. S10C). In all experiments, the direction of the observed responses generally reflected those obtained with recombinant protein.

VTN has been linked with migratory and proliferative effects (49) that are also associated with integrin activation (44). Accordingly, we assayed cells for VTN-associated alterations in these phenotypes. We observed no effect of VTN on the expression of EMT markers, chemotaxis toward VTN, or enhanced motility on a VTN-coated surface, although there was a modest increase in the proliferation of VTN-exposed DU145 and PC-3 under low-serum conditions (Supplementary Fig. S11). We also confirmed that the VTN-associated survival suggested by reduced hypodiploid events does not translate to chemically induced apoptosis.

Taken together, we report that adhesion assay results predominantly reflect cell attachment, in addition to a separate effect of VTN on hypodiploid accumulation, and that VTN-induced phenotypes are consistent across varying peptide homology and biological origin.

### VTN associates with greater liver metastatic burden

**Figure 7.**
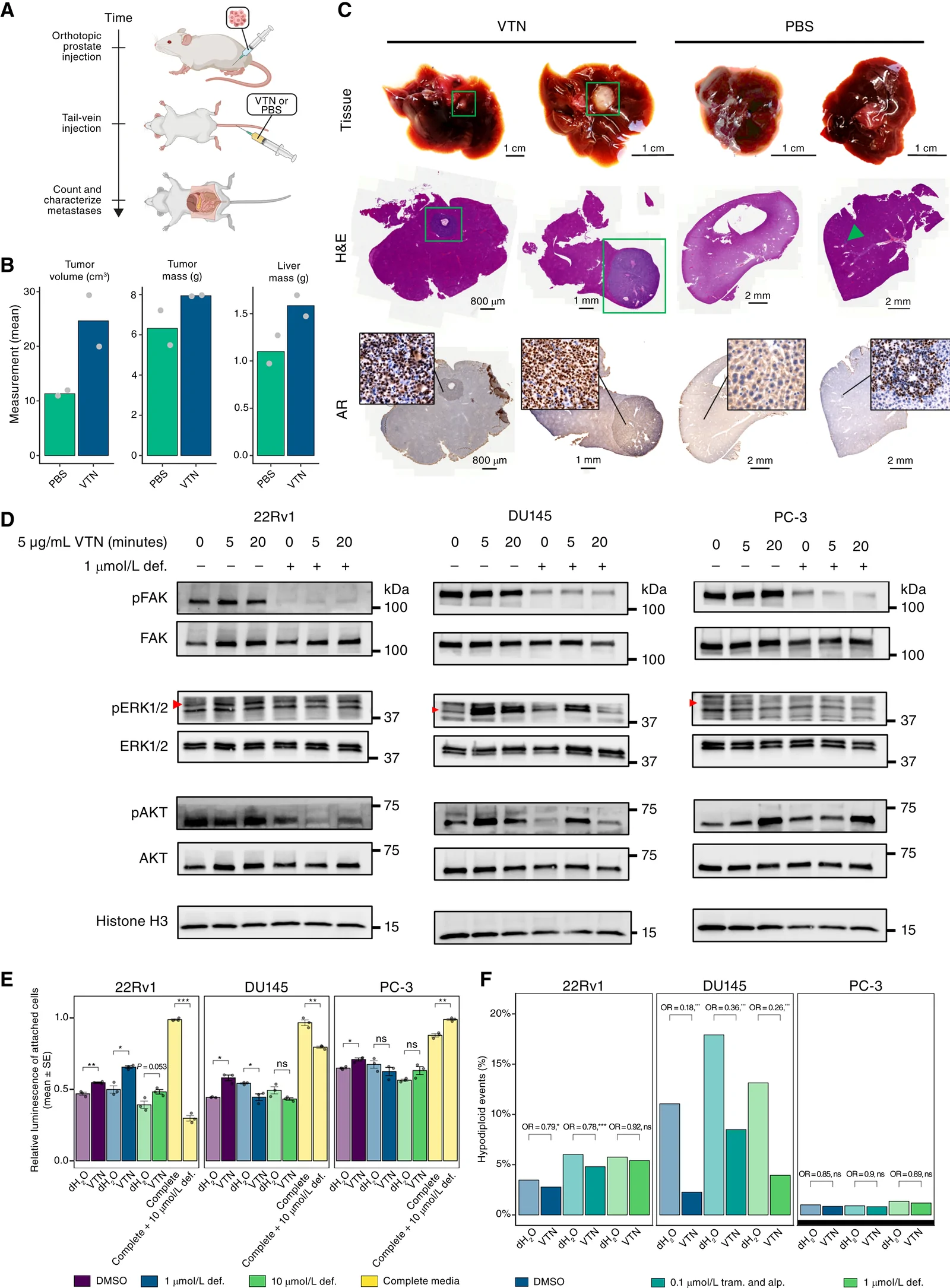
VTN is associated with greater liver metastatic burden, and its phenotypic effects may be mitigated by defactinib. **A,** Schematic of the *in vivo* experimental plan wherein mice undergo orthotopic 22Rv1 injection to the anterior prostate, tail-vein injections of VTN or PBS every other day, and necropsy to diagnose metastatic spread. **B,** Tumor volume, tumor mass, and liver mass were measured at necropsy. Results are plotted for two biological replicates. **C,** Whole livers were excised and examined for metastatic foci. Livers were chunked, formalin-fixed, paraffin-embedded, and hematoxylin and eosin (H&E)–stained before re-examination for micrometastases. Whole tissue with grossly visible metastatic foci highlighted in green and H&E of tissue regions with metastases and corresponding AR IHC are shown. **D,** Cells were plated and serum-starved overnight with or without 1 μmol/L defactinib. Then, cells were exposed to VTN and DMSO or VTN and defactinib for the specified amounts of time before cells were lysed, and immunoblotting was performed. Red triangles indicate the pERK bands. **E,** Adhesion assays were performed in serum-free media with the specified compounds and either 5 μg/mL VTN in dH_2_O or dH_2_O alone. Bars show three biological replicates, and significance was calculated with two-tailed Welch *t* tests. Complete, complete media used as a positive control; def., defactinib; ns, not statistically significant. **F,** Cells were plated in 6-well plates in serum-free media with either 5 μg/mL VTN in dH_2_O or dH_2_O alone, in the context of the MEK and PI3K inhibitors trametinib and alpelisib in combination or the FAK inhibitor defactinib, and allowed to adhere for 24 hours. Cells were harvested for PI staining followed by flow cytometry, and data were gated to exclude debris and doublets. Hypodiploid cell fractions were calculated as described in “Materials and Methods,” and statistical significance was determined from individual experiments with a two-tailed Fisher exact test. alp., alpelisib; tram., trametinib. *, *P* ≤ 0.05; **, *P* ≤ 0.01; ***, *P* ≤ 0.001.

Our lab previously showed that prostate orthotopic injection of 22Rv1 develops spontaneous liver metastases within 75 days (14). To explore whether systemic VTN exposure could influence prostate cancer liver metastasis *in vivo*, we employed this model in an exploratory murine cohort. 22Rv1 was injected into the anterior prostate of four mice, and two received tail-vein injections of 4 μg VTN in PBS, whereas the remaining received PBS alone (Fig. 7A). Treatment continued every other day for 54 days or 3 weeks prior to the previously documented time of metastasis.

At necropsy, we observed that VTN was associated with larger primary tumor volume and mass and that livers from VTN-treated mice weighed more than PBS-treated control livers (Fig. 7B). Further examination revealed grossly visible liver metastatic foci in the VTN group (*n* = 2/2) but not the PBS group (*n* = 0/2). However, histologic analysis later showed micrometastasis in one PBS-treated mouse liver measuring approximately 338 μm in maximal cross-section diameter (compared with diameters of 1,442 and 4,110 μm in VTN-treated mice; Fig. 7C). IHC confirmed nuclear AR positivity of all identified metastases.

Although limited by sample size, our observations provide preliminary evidence for an association between VTN exposure and *in vivo* prostate cancer metastatic burden that aligns with VTN’s sinusoidal localization and *in vitro* attachment, apparent prosurvival, and signaling effects.

### FAK inhibition can prevent VTN-mediated attachment and hypodiploid depletion

Considering these findings, we explored pharmaceutical compounds capable of inhibiting VTN-induced phenotypes. Prior work demonstrates that FAK, ERK, and AKT can contribute to cell adhesion and spreading (50–52), so we investigated whether FAK, MEK, or PI3K inhibition could abolish VTN-induced adhesion. We observed that FAK inhibition by defactinib impaired VTN-stimulated ERK and AKT phosphorylation in 22Rv1 and DU145 and ERK phosphorylation in PC-3 (Fig. 7D). Defactinib also prevented VTN-mediated adhesion in DU145 but not 22Rv1 (Fig. 7E). Neither the MEK inhibitor trametinib nor PI3K inhibitor alpelisib independently or in combination affected adhesion (Supplementary Fig. S12). However, we confirmed that trametinib and alpelisib successfully mitigated ERK and AKT phosphorylation in 22Rv1 and DU145 (Supplementary Fig. S13). None of the tested drugs affected post-adhesion cell spreading, but defactinib inhibited attachment of 22Rv1 and DU145 in complete media.

As combined trametinib and alpelisib treatment did not abolish VTN-mediated attachment, we hypothesized that ERK and AKT phosphorylation might influence hypodiploid accumulation instead. Therefore, we repeated PI staining with exposure to VTN, trametinib and alpelisib, or defactinib. In 22Rv1, VTN was associated with reduced hypodiploid accumulation under DMSO, whereas this within-condition difference was not detected in the presence of defactinib. In DU145, VTN was associated with reduced hypodiploid accumulation at baseline and following treatment with trametinib, alpelisib, or defactinib. However, measured effect sizes with trametinib and alpelisib or defactinib treatment were lower than those at baseline (Fig. 7F).

**Figure 8.**
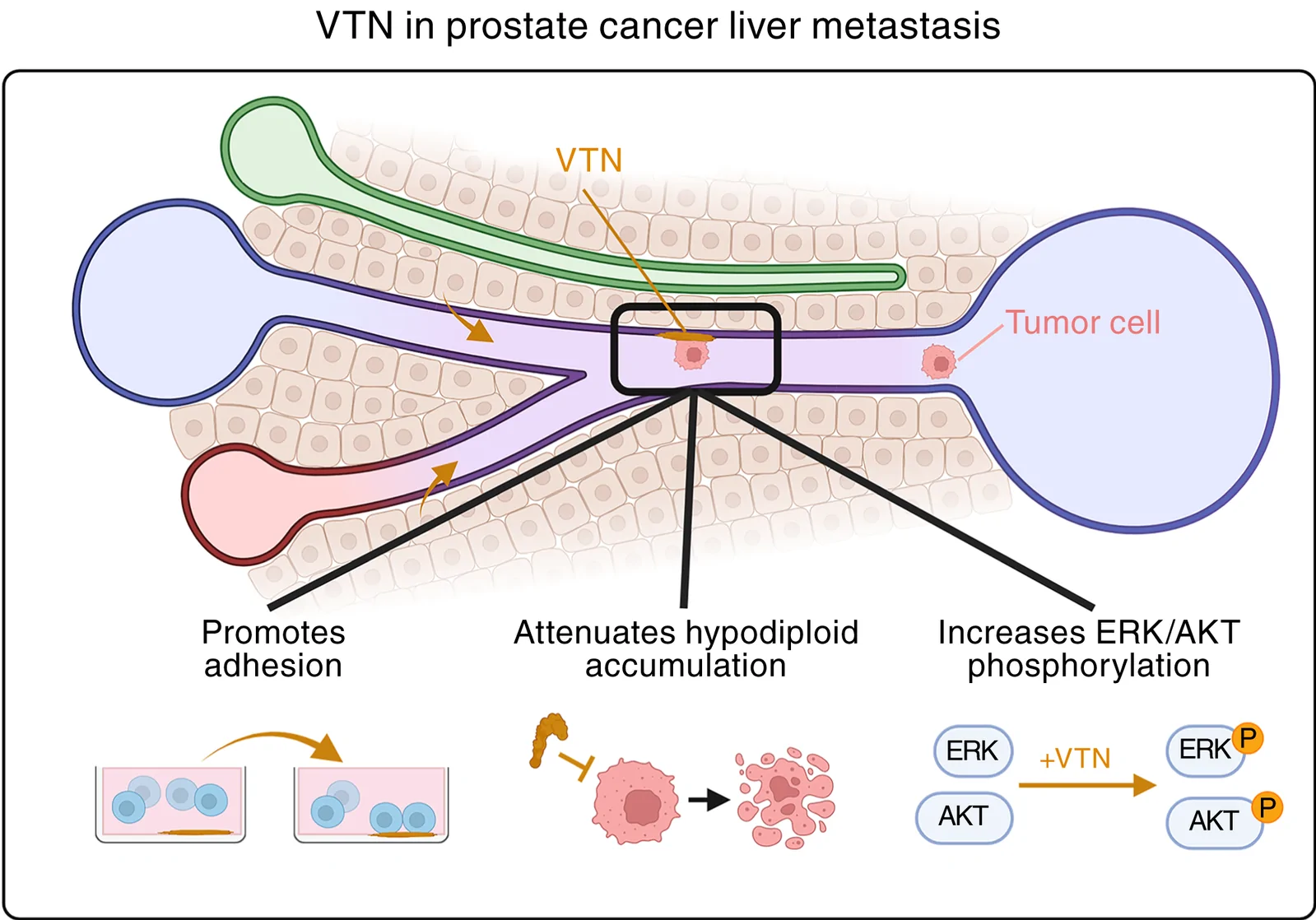
Schematic of the study findings. VTN accumulates along the hepatic sinusoid lumen, where current models of metastasis suggest it would encounter circulating tumor cells. Our results indicate that VTN promotes adhesion and spreading, inhibits hypodiploid accumulation in a manner consistent with prosurvival effects, and activates MAPK and PI3K in prostate cancer cells. [Created in BioRender. Egelberg, J. (2026) https://BioRender.com/xlzui2w.].

These results demonstrate that VTN exerts multimodal effects on prostate cancer cells (Fig. 8): (i) promoting adhesion to nearby surfaces, (ii) reducing apoptosis-associated hypodiploid events, and (iii) activating the MAPK and PI3K pathways. Taken together, the phenotypic and histopathologic observations reported here support a model wherein VTN physically interacts with and promotes the survival of prostate cancer in the liver microenvironment, which may be inhibited by defactinib in some models.

## Discussion

We identified VTN as a potential mediator of cell–cell interactions between hepatocytes and tumor cells promoting prostate cancer liver metastasis. Our analysis, across heterogeneous liver metastases spanning CRPC and NEPC, pointed to consistent activation of integrin signaling by VTN. Functionally in prostate cancer cell lines, VTN promotes cell adhesion, inhibits hypodiploid accumulation consistent with prosurvival effects, and induces FAK-dependent phosphorylation of ERK and AKT. Histologic analyses of prostate cancer liver metastases identified tumor cells surrounded by VTN-enriched hepatocytes and VTN accumulation in dense patches that line the sinusoid lumen. These observations support a model (Fig. 8) wherein prostate tumors physically interact with VTN-lined liver capillaries, allowing tumor cells to survive, colonize, and grow. This effect may be countered by FAK inhibition, as we found that defactinib mitigates both VTN-mediated and serum-mediated cell adhesion as well as the hypodiploid depletion effects of VTN exposure in a model-specific manner.

VTN could contribute to the clinical aggressiveness observed with liver metastases via diverse mechanisms. First, VTN concentrated at sinusoidal patches in the liver may provide a platform for cells to bind. Second, VTN might promote survival of circulating tumor cells and increase the odds of successful extravasation and metastasis. Third, VTN-activated integrins may cooperate with other growth factors and receptors to drive invasion, potentially through ERK- or AKT-related mechanisms (53).

Our results contribute to and align with prior studies implicating integrin–ECM interactions in organotropism (54). CellChat analysis of liver metastases predicted that the cell receptors to bind VTN include the integrins α_V_β_1_ and α_V_β_5_. Previous work has shown that α_V_ and α_5_β_1_ cross-talk promotes EMT-associated programs in prostate cancer (55) and that α_V_β_5_ is specifically and significantly enriched in liver-tropic exosomes (56). Existing research also implicates integrins and VTN particularly in prostate cancer bone metastasis; recovery of α_V_β_3_ expression in C4-2 cells significantly promotes tumorigenicity in bone and adhesion on VTN (57), which is deposited along skeletal muscle (58). Similar to our identification of dense intrasinusoidal VTN deposits, past research describes that similar depositions of fibronectin are utilized by CT26 colorectal carcinoma cells for endothelial adhesion (59).

Beyond VTN, our cell communication analysis of liver metastases pointed to other features of the tumor microenvironment that might contribute to tumor aggressiveness. This included Notch signaling, which can be oncogenic (60) or tumor suppressive (61). Of the three tumor clusters, only AR-enriched cluster Tumor_4 was predicted to receive incoming Notch signals by CellChat. This predicted communication consisted of the stimulatory Notch ligands JAG1 and JAG2, expressed by hepatocytes and tumor cells, binding to the NOTCH1, NOTCH2, and NOTCH3 receptors. Tumor_4 also significantly downregulated the inhibitory Notch ligand DLL3 relative to AR-depleted clusters Tumor_3 and Tumor_0. Interestingly, The Human Protein Atlas shows enhanced *JAG1* and *JAG2* expression in normal prostate tissue relative to normal liver tissue (FC = 4.2× and 8.6×, respectively). These results suggest that suppressed hepatocyte *JAG* expression may reduce tumor Notch activation and might contribute to the evolution of lineage plasticity (62).

Limitations of the current study complicate the interpretation of single-cell and cell communication analysis. First, a limited number of liver metastases have undergone single-cell RNA-seq. It is possible that some cell states which were not captured in the current data do not respond to VTN. Second, although CellChat predicted VTN to be a component of hepatocyte–tumor interactions, existing literature shows that VTN is secreted into the bloodstream and accumulates throughout the body. Our data emphasize this accumulation in sinusoids, but we do not compare secreted VTN quantities in the liver with other deposition sites, which encompass common regions of metastasis including the lungs and brain (58, 63). Third, the *in vivo* work presented here was exploratory and underpowered. Larger sample sizes are necessary to identify significant associations.

Together, our data highlight extensive heterogeneity among liver metastases and nominate FAK inhibition by defactinib as a novel therapeutic strategy for further study. Up to 25% of patients with CRPC develop liver metastasis (3), and due in part to diverse phenotypes spanning the full spectrum of disease, these lesions do not conform to current treatment paradigms. In May 2025, responding to positive clinical outcomes in the phase I FRAME (NCT03875820) and phase II RAMP-201 (NCT04625270) trials (64, 65), the FDA granted accelerated approval to defactinib in combination with the RAF–MEK inhibitor avutometinib for *KRAS*-mutated low-grade serous ovarian cancer. These trials did not include patients with prostate cancer or specifically report the impact of therapy on liver metastases.

Given our results, expanded *in vivo* studies should explore model-specific mechanisms of defactinib resistance and the ability of defactinib to mitigate liver metastasis in prostate cancer and potentially other cancer types, such as ovarian, colorectal, pancreatic, and breast cancers (15, 59, 66). Future research is also warranted to characterize the phenotypic effects of VTN-stimulated ERK and AKT phosphorylation, explore if and how VTN exposure affects lineage transitions in prostate cancer, and evaluate where VTN deposits accumulate in the body.

## Supplementary Material

Figure S1 Figure S1. Quality control of single-cell transcriptomic samples.

Figure S2 Figure S2. Pathological analysis of tumor cells.

Figure S3 Figure S3. VTN expression is highly hepatocyte-specific and prostate cell lines in DepMap express receptors for VTN with minimal expression of VTN itself.

Figure S4 Figure S4. Sensitivity, down-sampling, and generalizability analyses of inferred VTN communication.

Figure S5 Figure S5. Liver-metastatic models are associated with increased VTN-mediated adherence.

Figure S6 Figure S6. Propidium iodide staining enables quantification of hypodiploid cell fractions.

Figure S7 Figure S7. Basal pAKT signal is lower in 22Rv1 under tested conditions.

Figure S8 Figure S8. No consistent changes in YAP or β-catenin phosphorylation or NOTCH activity were observed following VTN stimulation.

Figure S9 Figure S9. VTN foci promote differential spreading of 22Rv1 and DU145 but not PC3.

Figure S10 Figure S10. Murine and human-derived VTN recapitulate recombinant VTN induced effects.

Figure S11 Figure S11. VTN exposure does not promote EMT-associated phenotypes.

Figure S12 Figure S12

Figure S13 Figure S13. Trametinib suppresses ERK phosphorylation, whereas alpelisib suppresses AKT phosphorylation in 22Rv1 and DU145; alpelisib does not reduce pAKT in PC-3.

Table S1 Table S1

Table S2 Table S2

Table S3 Table S3

Table S4 Table S4

Table S5 Table S5

Table S6 Table S6

Table S7 Table S7

Table S8 Table S8

## Data Availability

Single-cell transcriptomic data of clinical samples and bulk sequencing of orthotopically implanted 22Rv1 and liver-metastasized cells were retrieved from the NCBI Gene Expression Omnibus series GSE210358 (34, 35) and GSE211448 (14), respectively. Bulk RNA-seq of the SU2C–PCF International Dream Team metastatic prostate cancer cohort was retrieved from cBioPortal. Normal human hepatocyte single-cell sequencing was retrieved from GSE115469 (17). Other data are available upon request to the corresponding author.
