# Supplementary material for: Vitronectin Enrichment in Prostate Cancer Liver Metastases Promotes Adhesion and Survival
Source: Cancer Res Commun. 2026 Sep 17;6(9):2187–205. doi: 10.1158/2767-9764.CRC-26-0305 (PMC13583505; doi:10.1158/2767-9764.CRC-26-0305)
Supplement: Supplementary file 1 [file crc-26-0305_figure_s10_suppsf10.pdf]

Supplementary Fig. 10

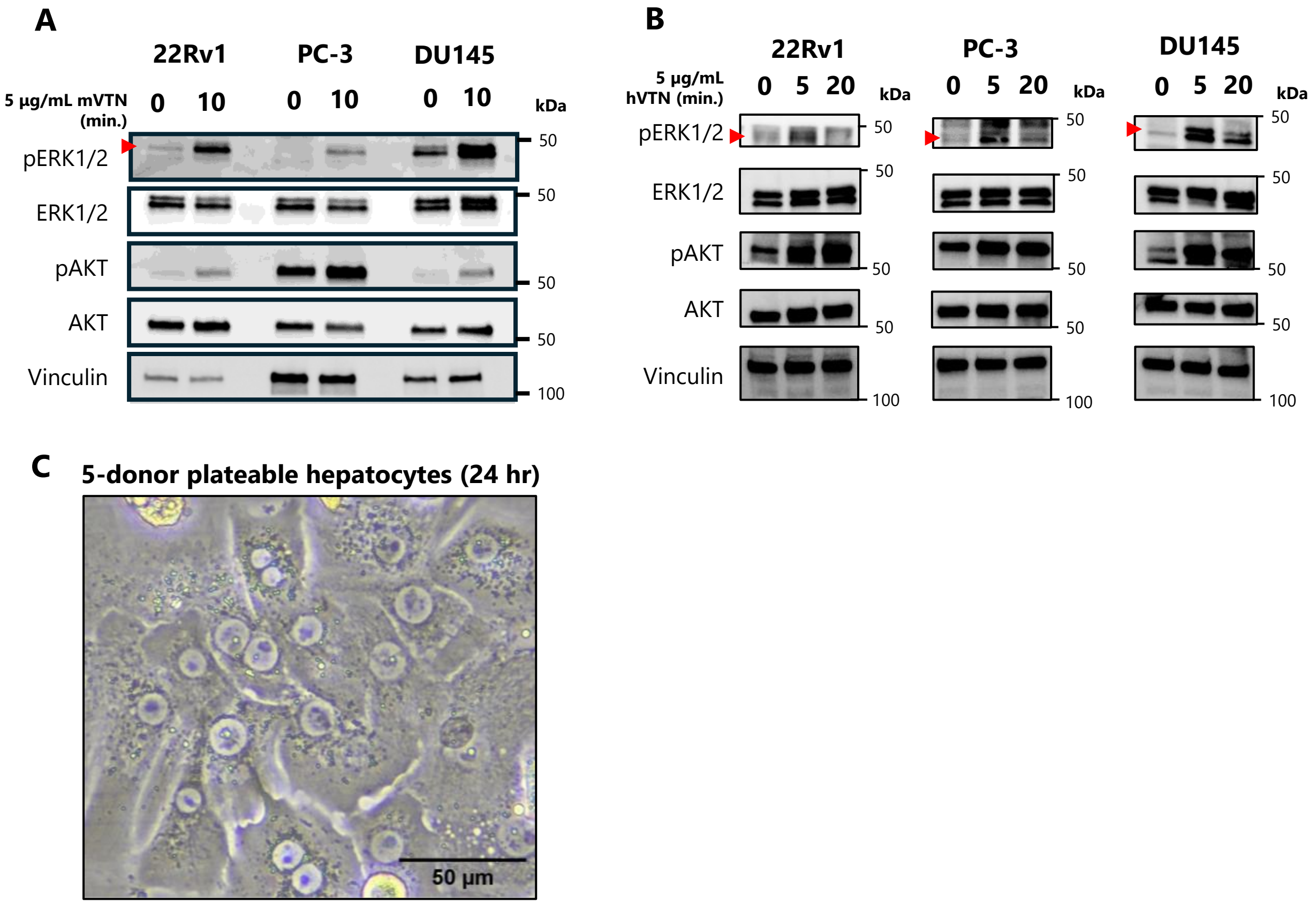

Figure S10. Murine and human-derived VTN recapitulate recombinant VTN-induced effects.

(**A**) Cells were plated on 6-well plates, serum starved overnight, and exposed to murine VTN for the specified amounts of time. After exposure, cells were lysed and immunoblotting was performed as specified. Red triangles indicate the pERK bands. (**B**) Cells were plated on 6-well plates, serum starved overnight, and exposed to human plasma-purified VTN for the specified amounts of time. After exposure, cells were lysed and immunoblotting was performed as specified. Red triangles indicate the pERK bands. (**C**) Representative image of 5-donor human plateable hepatocytes 24 hours after plating.
