# Supplementary material for: Vitronectin Enrichment in Prostate Cancer Liver Metastases Promotes Adhesion and Survival
Source: Cancer Res Commun. 2026 Sep 17;6(9):2187–205. doi: 10.1158/2767-9764.CRC-26-0305 (PMC13583505; doi:10.1158/2767-9764.CRC-26-0305)
Supplement: Supplementary file 2 [file crc-26-0305_figure_s11_suppsf11.pdf]

Supplementary Fig. 11

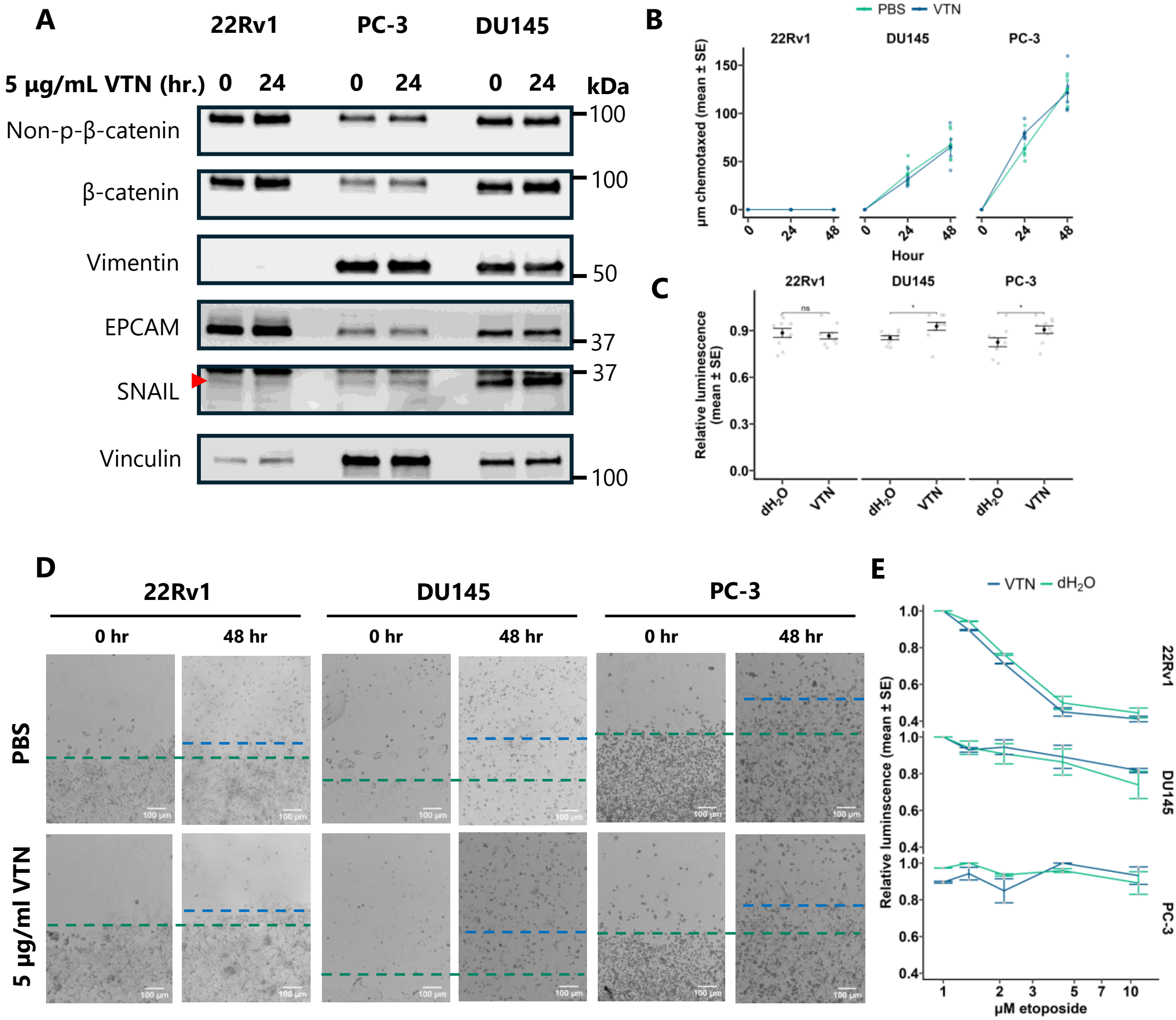

Figure S11. VTN exposure does not promote EMT-associated phenotypes.

(**A**) Cells were plated on 6-well plates, serum starved overnight, and exposed to VTN for the specified amounts of time. After exposure, cells were lysed and immunoblotting was performed as specified. Red triangles indicate the SNAIL band. (**B**) A gasket was pressed firmly against the center of each well in a 6-well plate. The region within the gasket was coated with 80 µL of 5 µg/mL VTN in PBS and 1.5 mL of cell-rich media was added around the gasket. The plate was placed at 37ºC until cells were confluent. The gasket center was washed with PBS, the gasket was removed, and cells were allowed to migrate into the coated region. Distance chemotaxed was calculated as the Euclidean distance from a fixed reference location to five points along the cell front from individual wells. Results are shown for a single experiment. (**C**) 5,000 cells were plated in 96-well plates and allowed to attach in complete media. Low serum (1%) media was replenished once daily with 5 µg/mL VTN in dH2O or dH2O alone for four days. Viable cells were quantified with CellTiter-Glo and two-tailed Welch's t-tests were used for determinations of statistical significance. Results are shown for 10 biological replicates. (**D**) 6-well plates were coated with 2 mL 5 µg/mL VTN in PBS or PBS alone as described. Then, a gasket was pressed firmly against the center of each well and 1.5 mL of cell-rich media was added around the gasket. The plate was placed at 37ºC until cells were confluent. The gasket was removed and cells were allowed to migrate into the center. Dotted lines were drawn to annotate the cell front with green indicating the front at the time of plating and blue indicating the front 48 hours later. (**E**) 20,000 cells were plated in 96-well plates in complete media and allowed to adhere overnight. Media with etoposide and either 5 µg/mL VTN in dH2O or dH2O alone was added for 72 hours before cells were washed once with PBS and viability was quantified with CellTiter-Glo. Results are shown for two biological replicates.
