# Supplementary material for: Vitronectin Enrichment in Prostate Cancer Liver Metastases Promotes Adhesion and Survival
Source: Cancer Res Commun. 2026 Sep 17;6(9):2187–205. doi: 10.1158/2767-9764.CRC-26-0305 (PMC13583505; doi:10.1158/2767-9764.CRC-26-0305)
Supplement: Supplementary file 3 [file crc-26-0305_figure_s12_suppsf12.pdf]

Supplementary Fig. 12

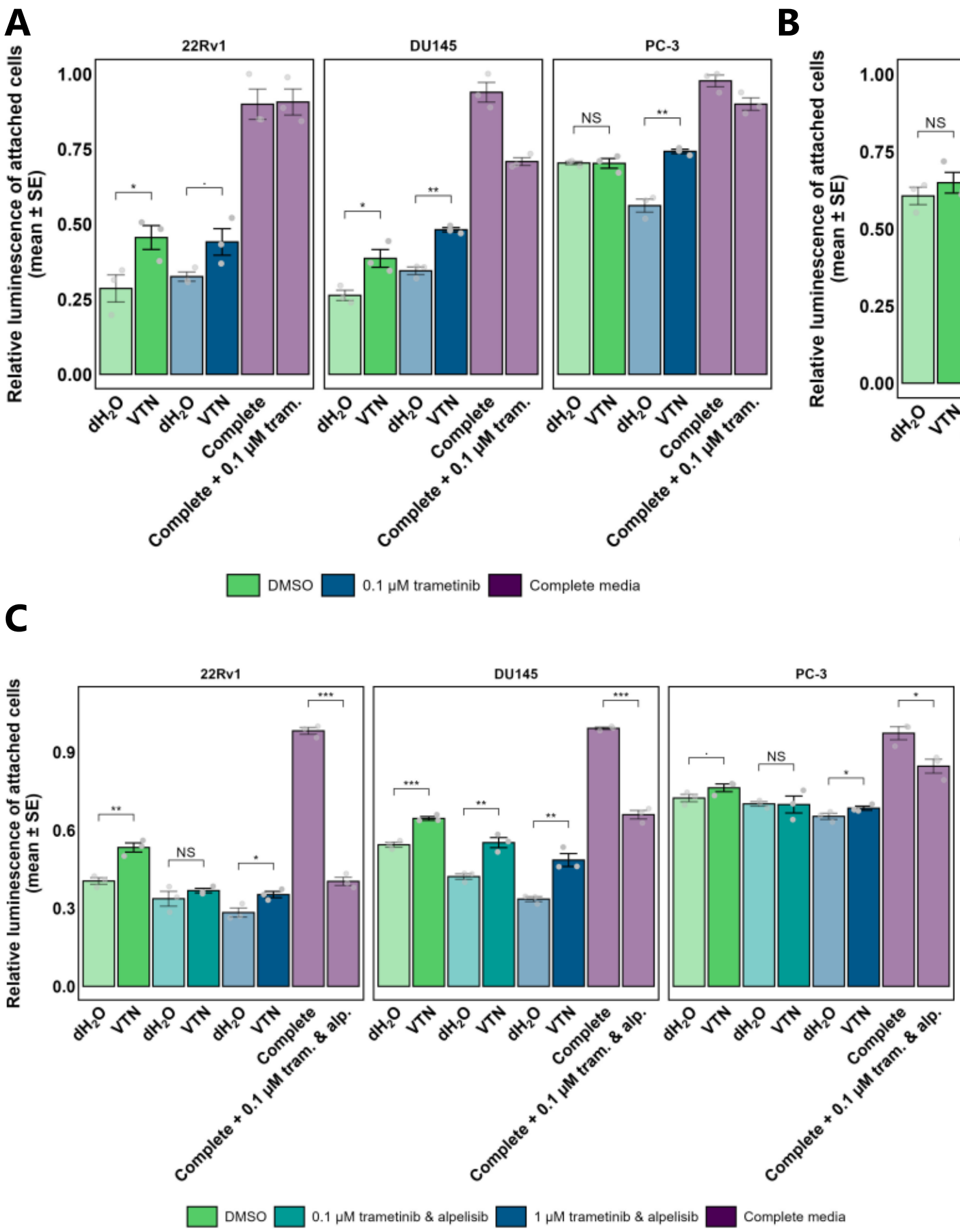

Figure S12. Neither trametinib nor alpelisib, alone or in combination, inhibits VTN-mediated attachment in 22Rv1 or DU145; effects in PC-3 are variable.

(**A-C**) Adhesion assays were performed in serum-free media with the specified compounds. Complete indicates the use of complete media as a positive control. Points show three biological replicates and significance was calculated with two-tailed Welch's t-tests.
