# Supplementary material for: Vitronectin Enrichment in Prostate Cancer Liver Metastases Promotes Adhesion and Survival
Source: Cancer Res Commun. 2026 Sep 17;6(9):2187–205. doi: 10.1158/2767-9764.CRC-26-0305 (PMC13583505; doi:10.1158/2767-9764.CRC-26-0305)
Supplement: Supplementary file 4 [file crc-26-0305_figure_s13_suppsf13.pdf]

Supplementary Fig. 13

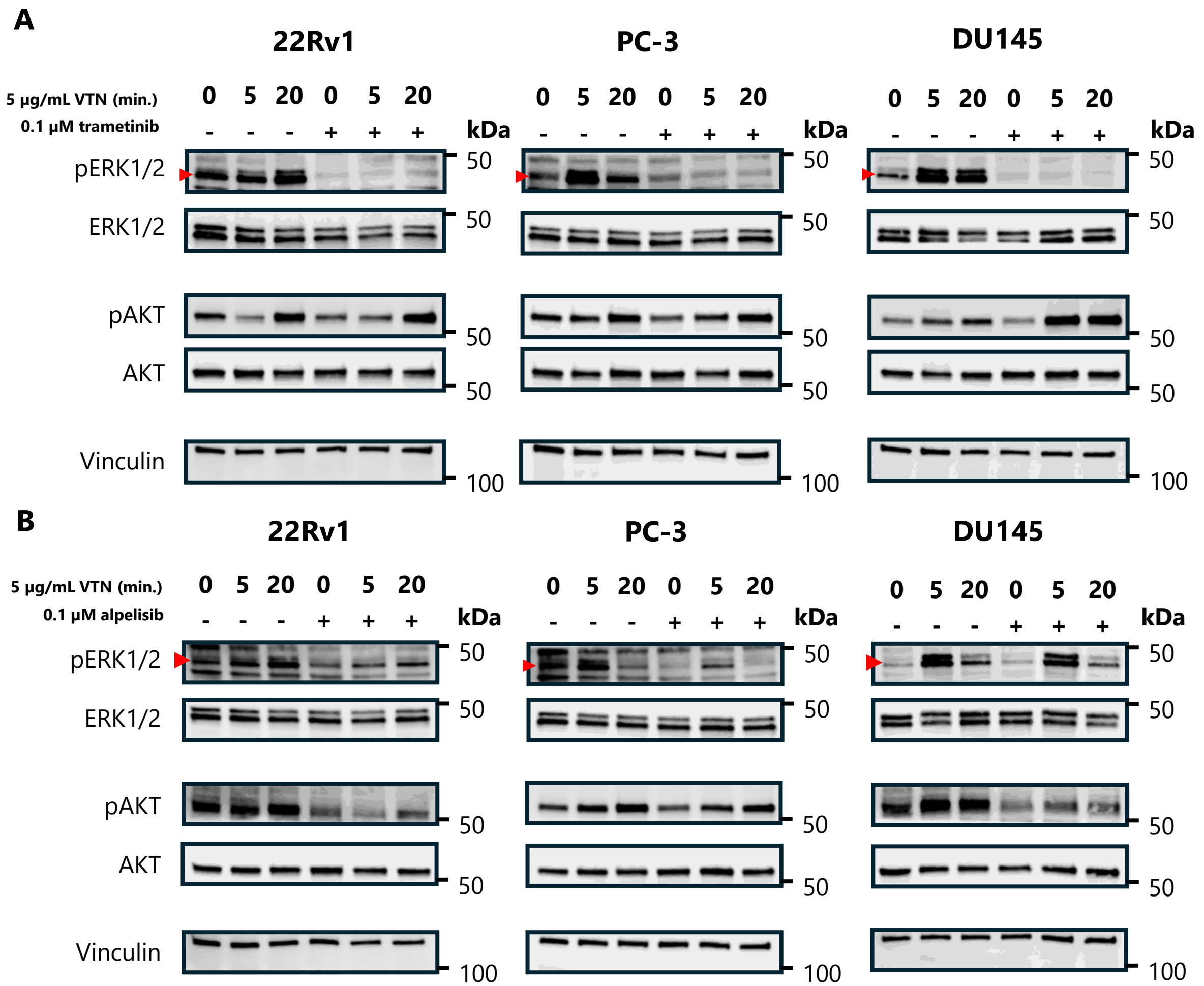

Figure S13. Trametinib suppresses ERK phosphorylation, whereas alpelisib suppresses AKT phosphorylation in 22Rv1 and DU145; alpelisib does not reduce pAKT in PC-3.

(**A-B**) Cells were plated on 6-well plates, serum starved overnight, and exposed to VTN for the specified amounts of time with indicated compound. After exposure, cells were lysed and immunoblotting was performed as specified. Red triangles indicate the pERK bands.
