# Supplementary material for: Vitronectin Enrichment in Prostate Cancer Liver Metastases Promotes Adhesion and Survival
Source: Cancer Res Commun. 2026 Sep 17;6(9):2187–205. doi: 10.1158/2767-9764.CRC-26-0305 (PMC13583505; doi:10.1158/2767-9764.CRC-26-0305)
Supplement: Supplementary file 5 [file crc-26-0305_figure_s1_suppsf1.pdf]

Supplementary Fig. 1

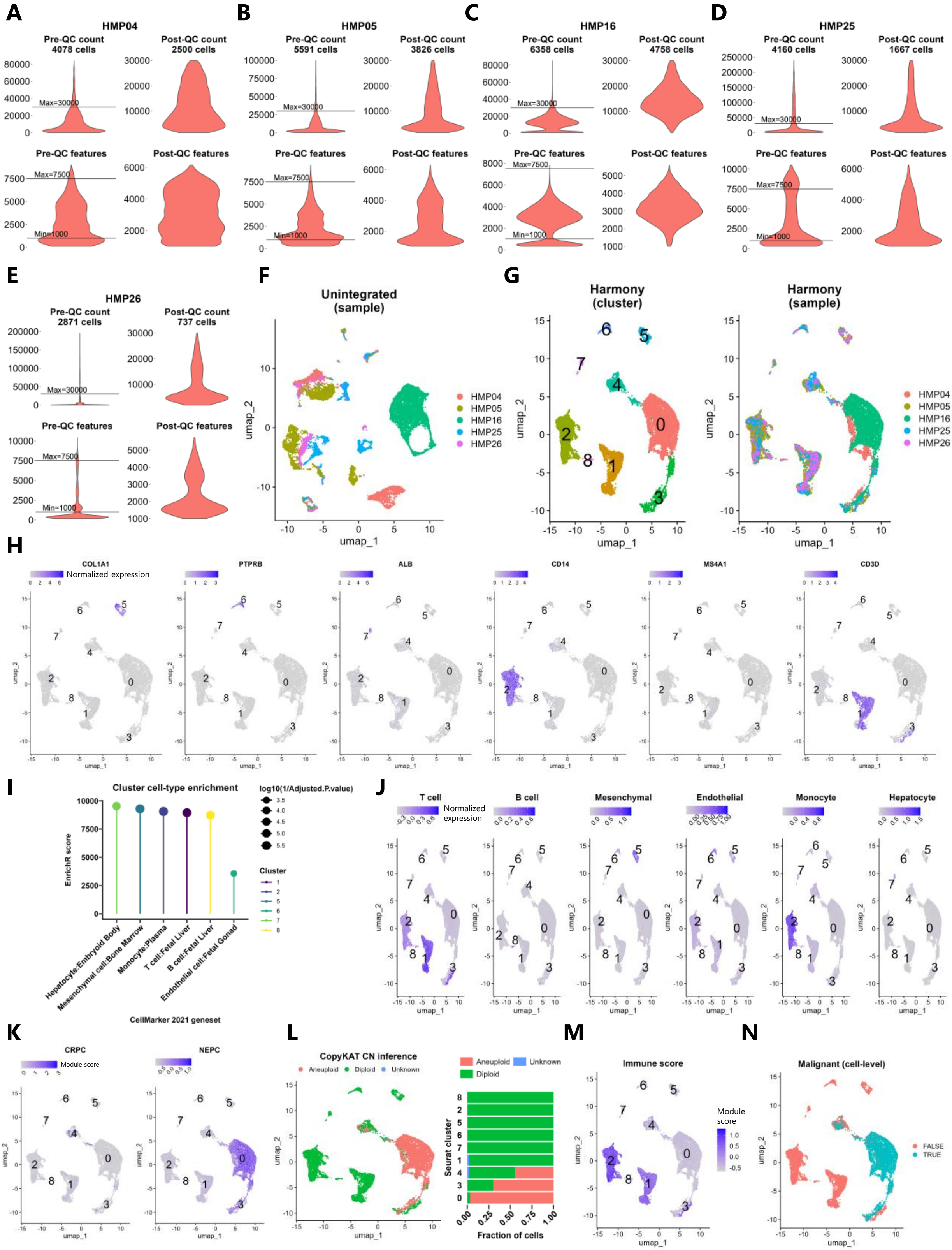

Figure S1. Quality control of single-cell transcriptomic samples.

(**A-E**) Counts and unique genes per cell prior to and post-quality control filtering. (**F**) Samples were merged into a single Seurat object, log-normalized, annotated with highly variable genes, scaled and centered, and analyzed for principal components. These components were used to construct a neighborhood graph and cluster like-transcriptomic states prior to 2-dimensional projection with UMAP. The final projection was colored by sample/patient for plotting. (**G**) Pre-processing was repeated but with Harmony integration prior to UMAP. Left: Harmony-integrated UMAP colored by Seurat cluster (resolution = 0.15). Right: Harmony-integrated UMAP colored by sample/patient. (**H**) Gene expression projected onto the Harmony-integrated UMAP for manually curated cell-type markers. (**I**) The top four upregulated cluster markers for each Harmony-integrated Seurat cluster were inputted to enrichR for gene list enrichment analysis against the CellMarker_Augmented_2021 gene set library. Shown are representative significantly enriched hits for each cluster. (**J**) Module scores for selected CellMarker_Augmented_2021 gene sets were calculated and projected onto the Harmony-integrated UMAP. (**K**) Module Scores for CRPC and NEPC gene sets were calculated and projected onto the UMAP. (**L**) CopyKAT was run on each sample with default parameters except for sample HMP16, which required ngene.chr=1 for model convergence. Left: CopyKAT cell-level predictions of copy number profile were projected onto the UMAP. Right: fraction of cells in each cluster with the shown copy number profile. (**M**) For each cell, an immune score was calculated by summing T Cell, B Cell, and Monocyte module scores. The total immune score was projected onto the UMAP for plotting. (**N**) Malignant cells were defined as cells belonging to clusters with large non-diploid populations (0, 3, or 4) with an immune score less than the median.
