# Supplementary material for: Vitronectin Enrichment in Prostate Cancer Liver Metastases Promotes Adhesion and Survival
Source: Cancer Res Commun. 2026 Sep 17;6(9):2187–205. doi: 10.1158/2767-9764.CRC-26-0305 (PMC13583505; doi:10.1158/2767-9764.CRC-26-0305)
Supplement: Supplementary file 6 [file crc-26-0305_figure_s2_suppsf2.pdf]

Supplementary Fig. 2

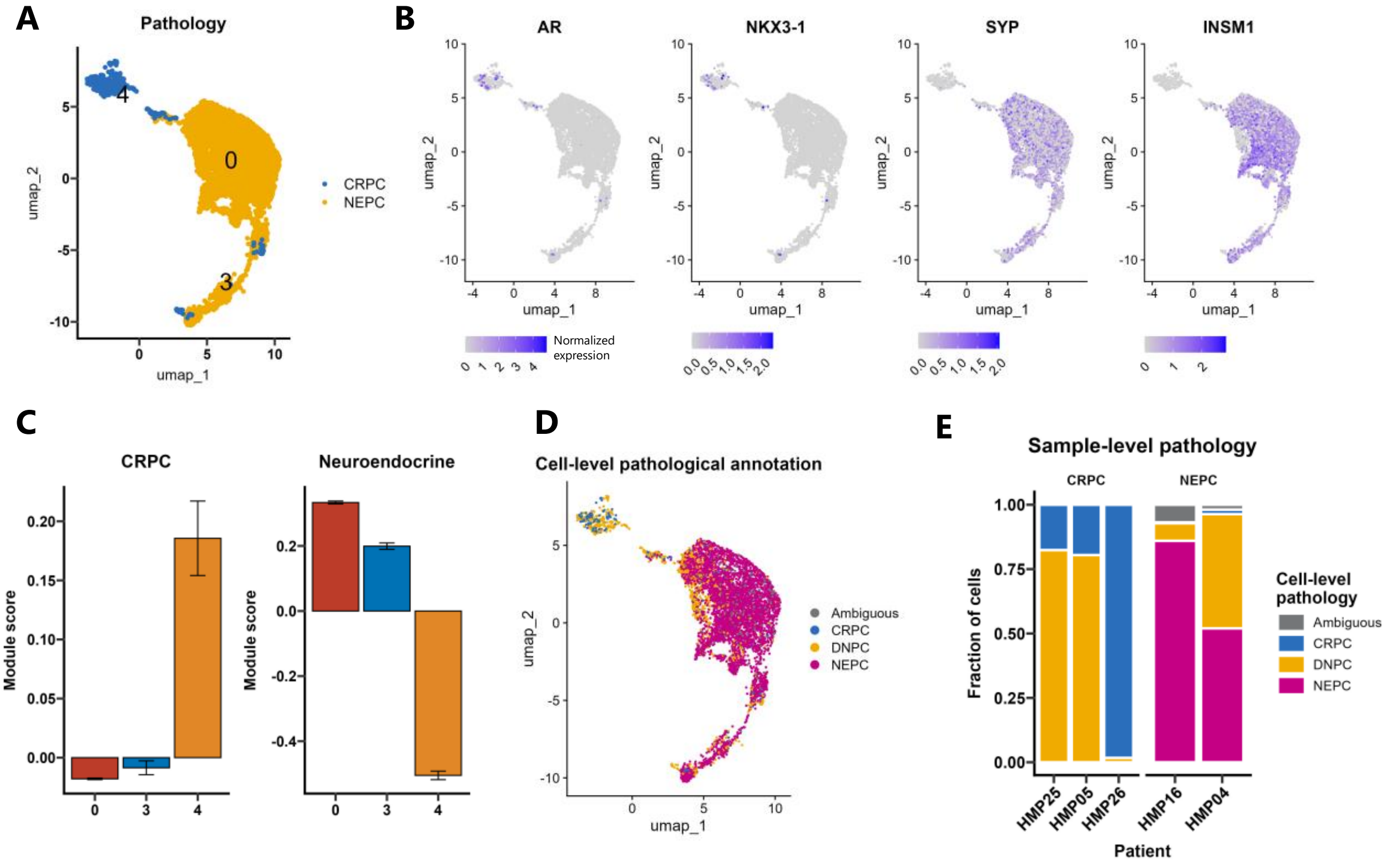

Figure S2. Pathological analysis of tumor cells.

(**A**) The parent Seurat object was subset to tumor cells and colored by sample-level pathology. (**B**) Expression of canonical adenocarcinoma and neuroendocrine markers was projected onto the UMAP. (**C**) CRPC (left) and neuroendocrine (right) module scores were averaged for tumor cells within each predominantly tumor cluster. (**D**) Cells were assigned to pathological state by CRPC and NEPC module scoring as follows: NEPC = NEPC > 0 & CRPC < 0; CRPC = NEPC < 0 & CRPC > 0; DNPC = NEPC < 0 & CRPC < 0; ambiguous= NEPC > 0 & CRPC > 0. Cell pathology was projected onto the UMAP for plotting. (**E**) Patients were facetted by sample-annotated pathology and filled by cell-level pathology derived from module scoring.
