# Supplementary material for: Vitronectin Enrichment in Prostate Cancer Liver Metastases Promotes Adhesion and Survival
Source: Cancer Res Commun. 2026 Sep 17;6(9):2187–205. doi: 10.1158/2767-9764.CRC-26-0305 (PMC13583505; doi:10.1158/2767-9764.CRC-26-0305)
Supplement: Supplementary file 7 [file crc-26-0305_figure_s3_suppsf3.pdf]

Supplementary Fig. 3

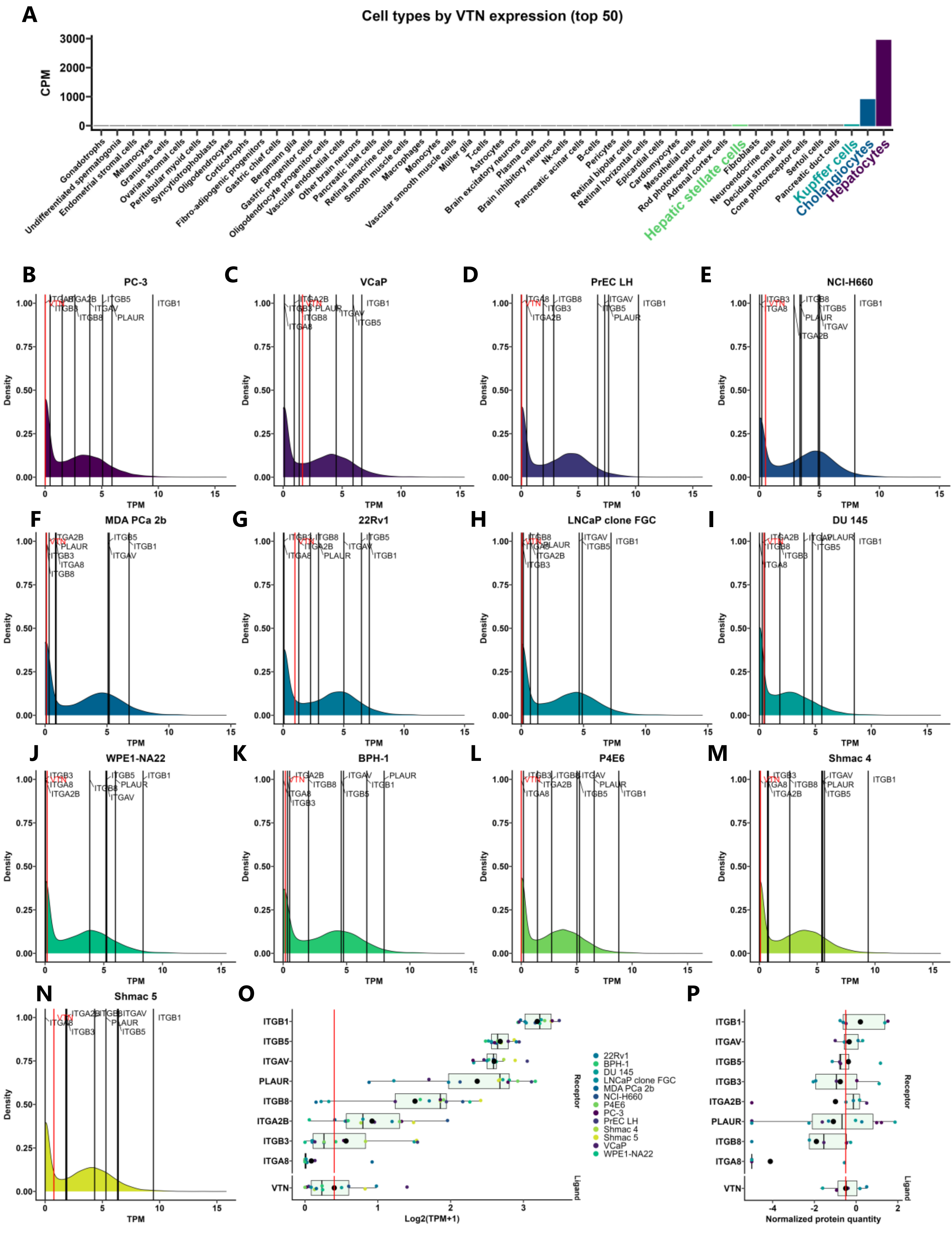

Figure S3. VTN expression is highly hepatocyte-specific and prostate cell lines in DepMap express receptors for VTN with minimal expression of VTN itself.

(**A**) Human Protein Atlas-aggregated single cell transcriptomic data pre-classified by cell type was examined for VTN expression. The top 50 VTN-expressing cell types are shown ordered by VTN expression and liver-resident cell types are colored and bolded. (**B-N**) Density plots of each DepMap prostate cell line from expression of all genes with black vertical lines highlighting VTN receptors and a red vertical line for VTN. (**O**) Expression of each VTN receptor and VTN is shown for all DepMap prostate cell lines. Mean expression was calculated for each gene across all cell lines (black points) and determined receptor ordering. The red vertical line denotes mean VTN expression. (**P**) Relative total protein for each VTN receptor and VTN is shown for profiled DepMap prostate cell lines. Mean protein quantity was calculated for each protein across all cell lines (black points) and determined receptor ordering. The red vertical line denotes mean VTN quantity.
