# Supplementary material for: Vitronectin Enrichment in Prostate Cancer Liver Metastases Promotes Adhesion and Survival
Source: Cancer Res Commun. 2026 Sep 17;6(9):2187–205. doi: 10.1158/2767-9764.CRC-26-0305 (PMC13583505; doi:10.1158/2767-9764.CRC-26-0305)
Supplement: Supplementary file 8 [file crc-26-0305_figure_s4_suppsf4.pdf]

Supplementary Fig. 4

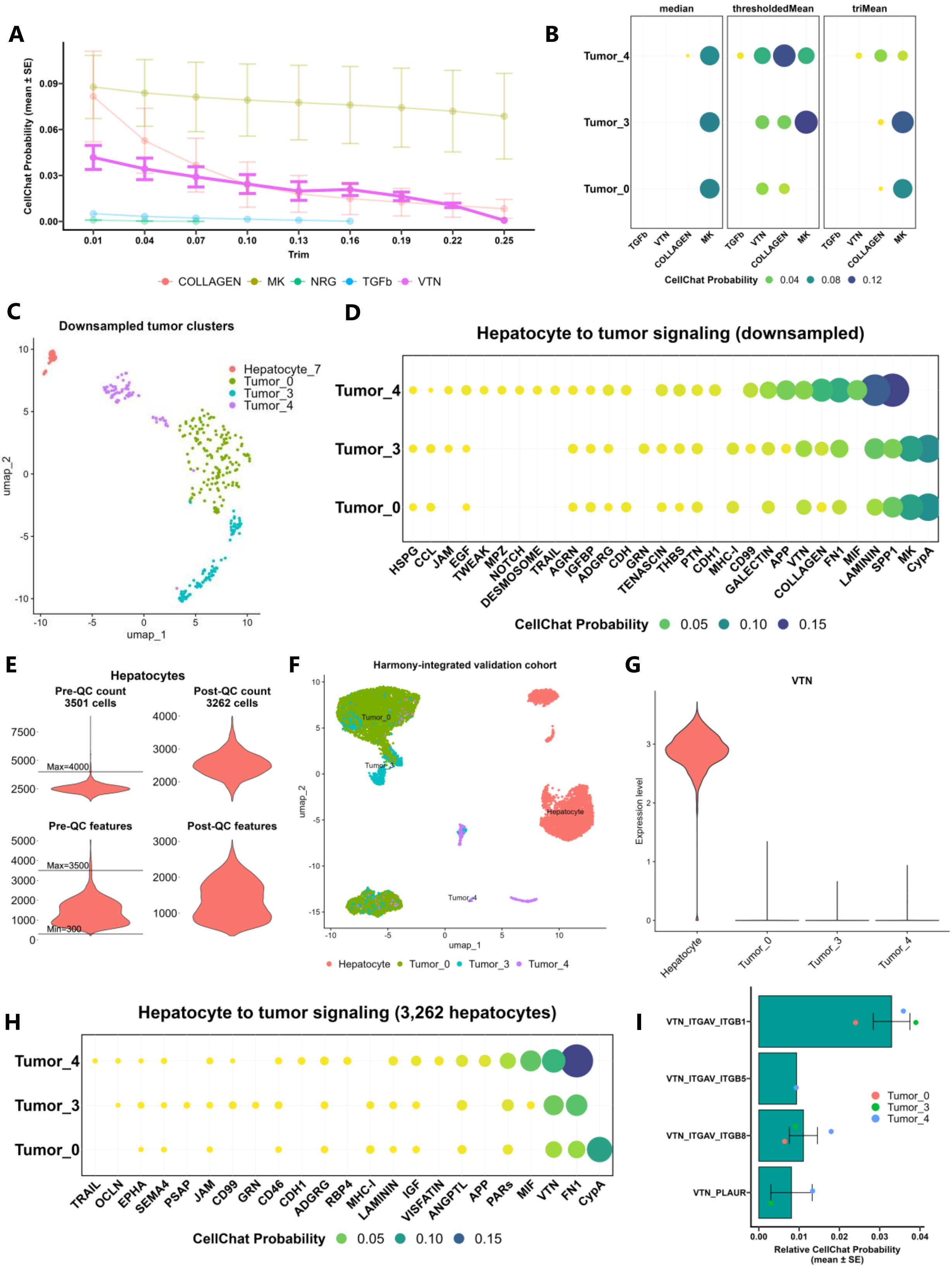

Figure S4. Sensitivity, down-sampling, and generalizability analyses of inferred VTN communication

(**A**) Trim values between 0 and 0.25 (the minimum and maximum) were inputted to CellChat (with the truncatedMean summary statistic) for communication inference. Predicted signaling was filtered to originate from hepatocytes and be received by tumor clusters and mean CellChat Probability was calculated across recipient tumor cluster. Predicted VTN communication with four additional pathways, representative of highly robust (MK), robust (COLLAGEN), sensitive (TGFb), and highly sensitive (NRG) signaling, are plotted. (**B**) The expression aggregation metric used during CellChat inference was permuted. Hepatocyte-to-tumor predicted signaling results are plotted for the same subset of communication pathways. (**C**) Tumor clusters were randomly sampled (seed=2) such that per-cluster tumor cell quantity equaled the quantity of hepatocytes (n=154). Down-sampled cells were plotted with UMAP. (**D**) CellChat was applied to infer communication between down-sampled tumor cells and hepatocytes with the same parameters as used in the initial/primary analysis (type="truncatedMean", trim=0.1). (**E**) Single-cell transcriptomic data obtained from GSE115469 was subset to pre-annotated hepatocytes and quality-controlled. (**F**) Curated hepatocytes were merged with tumor cells and re-integrated with Harmony, preserving initial annotations. (**G**) Normalized VTN expression was compared across the larger hepatocyte cohort and tumor cells. (**H**) CellChat was applied to infer hepatocyte-tumor communication using GSE115469-annotated hepatocytes (type="truncatedMean", trim=0.1). (**I**) Inferred VTN-binding receptors reflect tumor cell expression patterns and are identical between the larger and smaller hepatocyte cohorts.
