# Supplementary material for: Vitronectin Enrichment in Prostate Cancer Liver Metastases Promotes Adhesion and Survival
Source: Cancer Res Commun. 2026 Sep 17;6(9):2187–205. doi: 10.1158/2767-9764.CRC-26-0305 (PMC13583505; doi:10.1158/2767-9764.CRC-26-0305)
Supplement: Supplementary file 9 [file crc-26-0305_figure_s5_suppsf5.pdf]

Supplementary Fig. 5

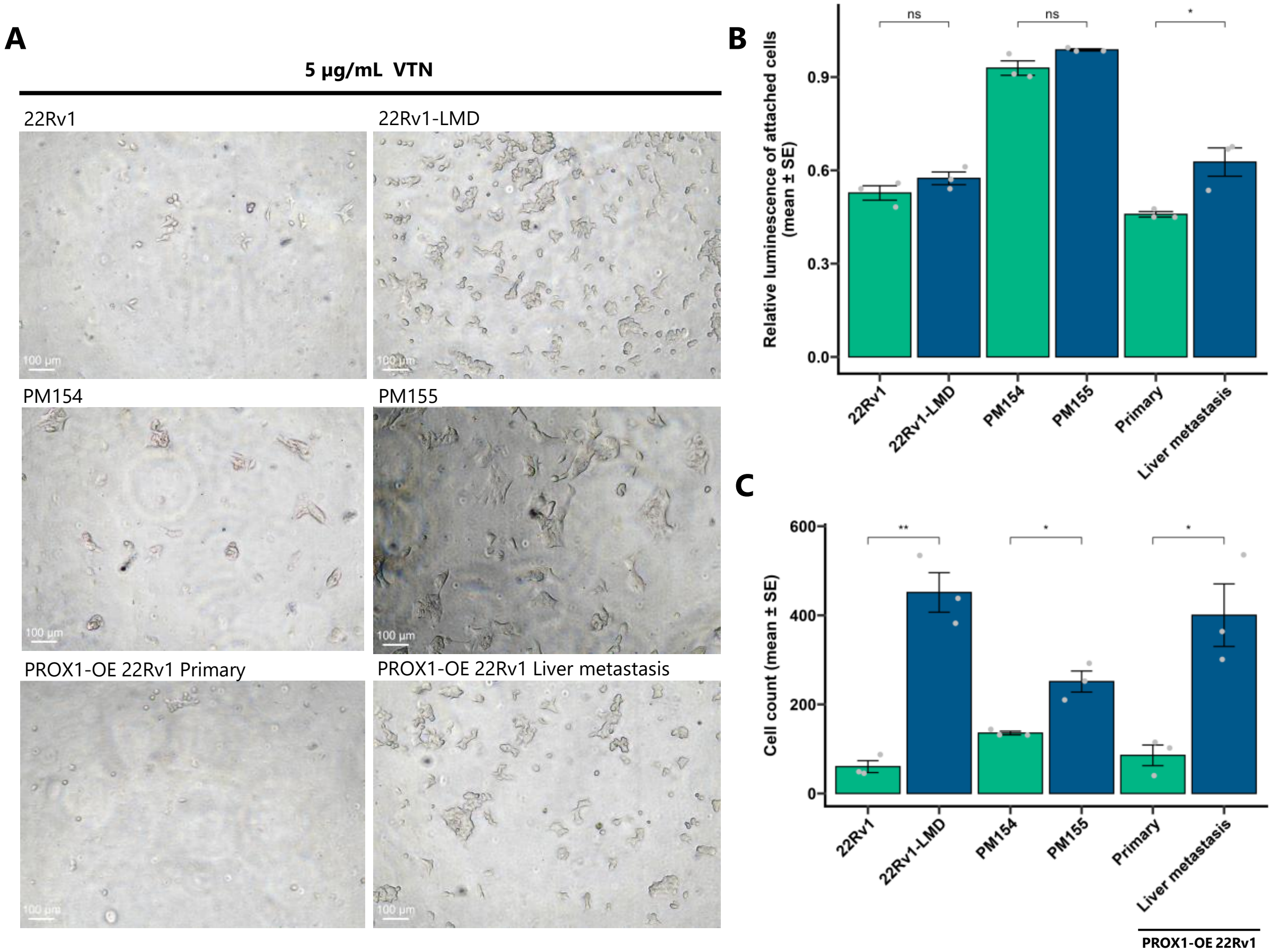

Figure S5. Liver-metastatic models are associated with increased VTN-mediated adherence.

(**A**) Images were taken of attached cells prior to CellTiter-Glo quantification. Shown are representative images depicting VTN-associated morphological transitions and differences in cell quantity between liver-metastatic models and their pair-matched controls following VTN exposure. (**B**) CellTiter-Glo luminescence was compared between liver-metastatic models and their pair-matched controls. Bars show three biological replicates and significance was calculated with two-tailed Welch's t-tests. (**C**) Cells from representative images taken across three biological replicates were manually counted and compared between models. Significance was calculated with two-tailed Welch's t-tests.
