# Supplementary material for: Vitronectin Enrichment in Prostate Cancer Liver Metastases Promotes Adhesion and Survival
Source: Cancer Res Commun. 2026 Sep 17;6(9):2187–205. doi: 10.1158/2767-9764.CRC-26-0305 (PMC13583505; doi:10.1158/2767-9764.CRC-26-0305)
Supplement: Supplementary file 10 [file crc-26-0305_figure_s6_suppsf6.pdf]

Supplementary Fig. 6

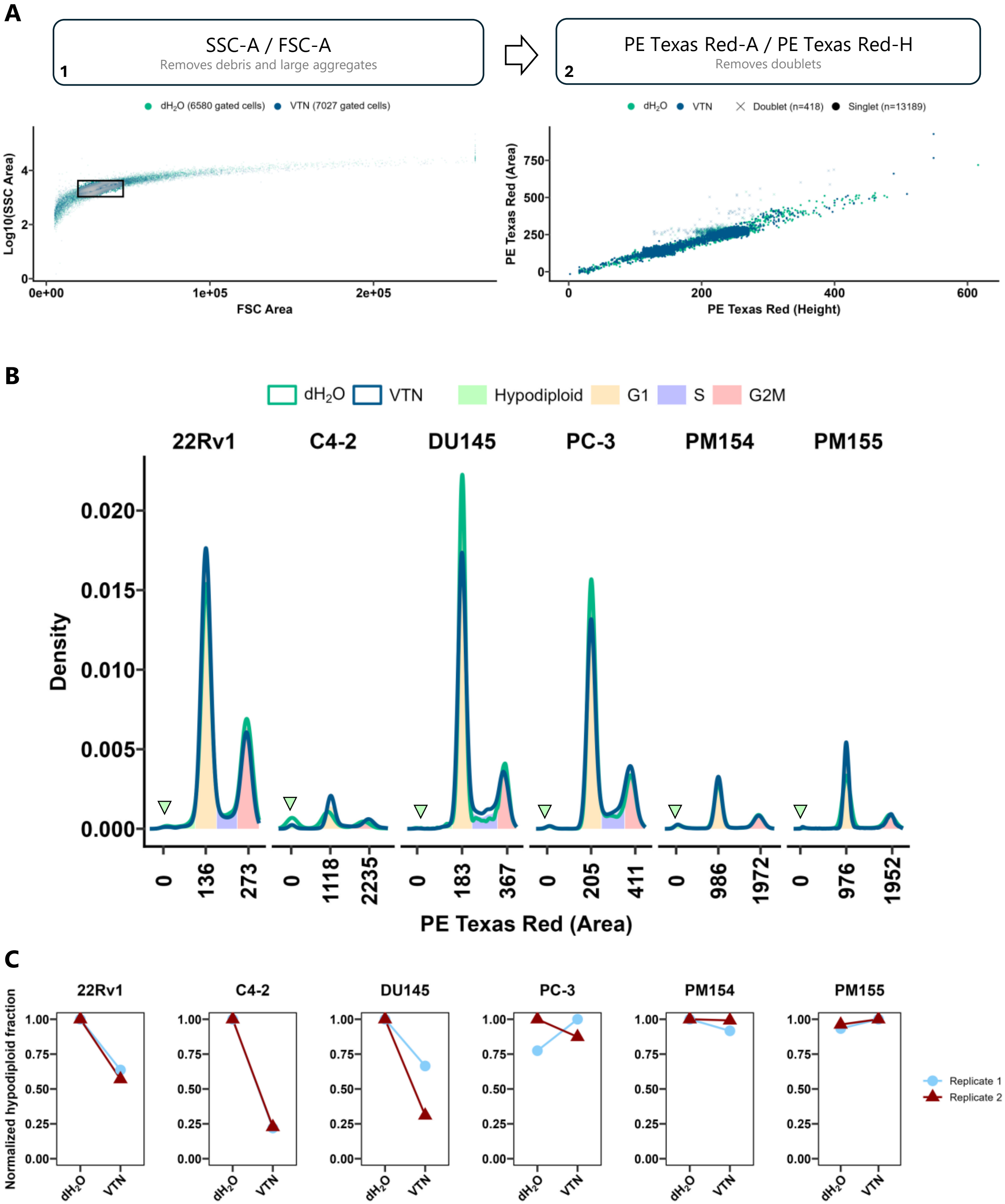

Figure S6. Propidium iodide staining enables quantification of hypodiploid cell fractions.

(**A**) Gating procedure for quantification of propidium iodide staining. SSC-A by FSC-A was used to exclude debris and large aggregates and PE Texas Red-A by PE Texas Red-H was used to exclude doublets. Representative gating of 22Rv1 cells is shown. (**B**) Densitometric curves of singlet-gated propidium iodide intensity captured by the PE Texas Red channel after 561 nm excitation. Green arrows point to hypodiploid cells. (**C**) Hypodiploid fractions from two biological replicate experiments were calculated for each cell line with and without VTN. Fractions were normalized to the maximum for each cell line and experiment and plotted. Results were consistent between experiments.
