# Supplementary material for: Vitronectin Enrichment in Prostate Cancer Liver Metastases Promotes Adhesion and Survival
Source: Cancer Res Commun. 2026 Sep 17;6(9):2187–205. doi: 10.1158/2767-9764.CRC-26-0305 (PMC13583505; doi:10.1158/2767-9764.CRC-26-0305)
Supplement: Supplementary file 11 [file crc-26-0305_figure_s7_suppsf7.pdf]

Supplementary Fig. 7

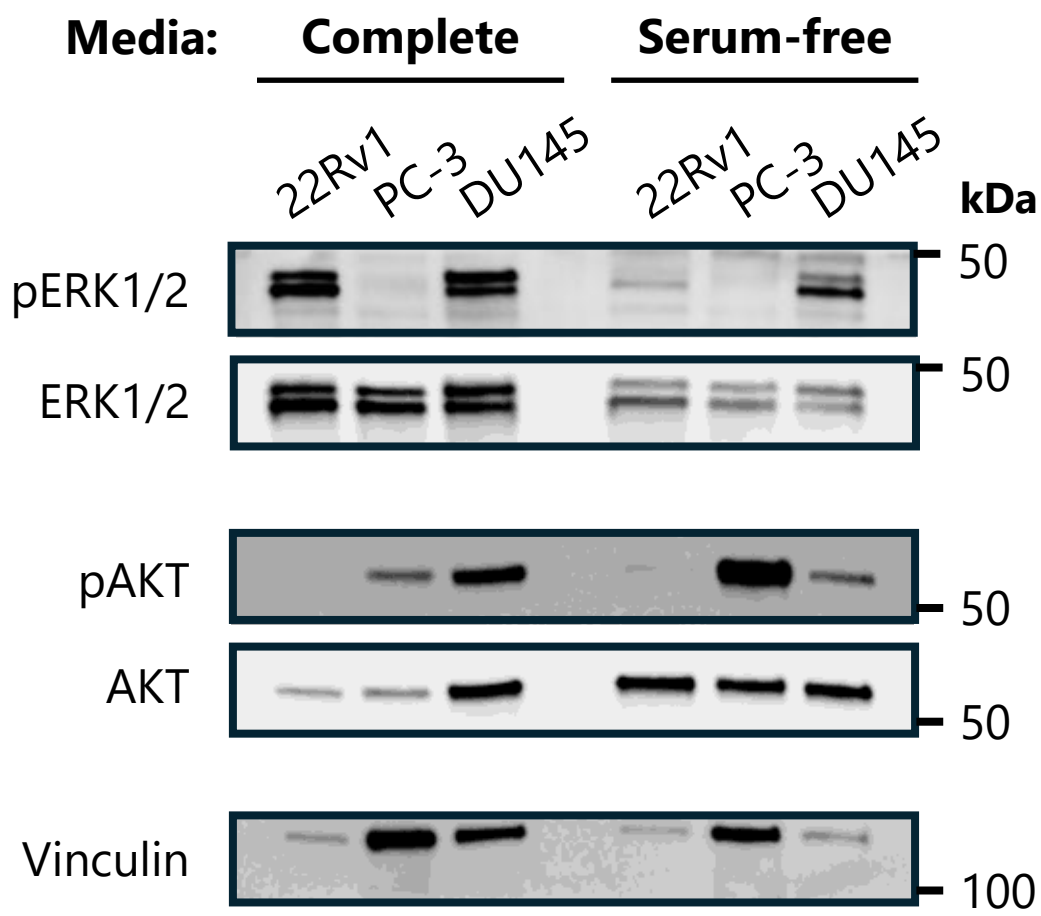

Figure S7. Basal pAKT signal is lower in 22Rv1 under tested conditions.

Cells were plated on 6-well plates in serum-free or complete media overnight. After 24 hours, cells were lysed and immunoblotting was performed as specified.
