# Supplementary material for: Vitronectin Enrichment in Prostate Cancer Liver Metastases Promotes Adhesion and Survival
Source: Cancer Res Commun. 2026 Sep 17;6(9):2187–205. doi: 10.1158/2767-9764.CRC-26-0305 (PMC13583505; doi:10.1158/2767-9764.CRC-26-0305)
Supplement: Supplementary file 12 [file crc-26-0305_figure_s8_suppsf8.pdf]

Supplementary Fig. 8

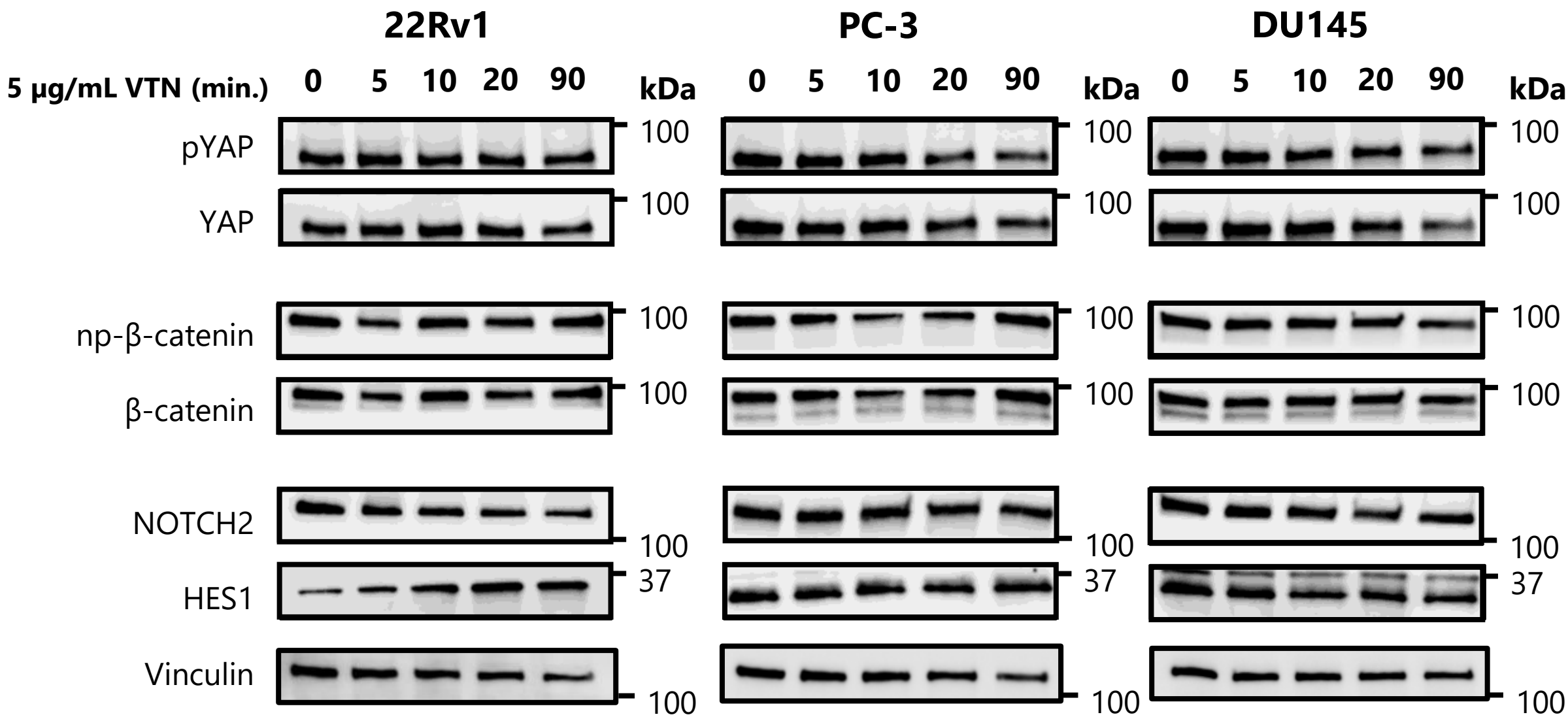

Figure S8. No consistent changes in YAP or β-catenin phosphorylation or NOTCH activity were observed following VTN stimulation.

Cells were plated on 6-well plates, serum starved overnight, and exposed to VTN for the specified amounts of time. After exposure, cells were lysed and immunoblotting was performed as specified. Non-phospho-β-catenin is indicated by np-β-catenin.
