# Supplementary material for: Vitronectin Enrichment in Prostate Cancer Liver Metastases Promotes Adhesion and Survival
Source: Cancer Res Commun. 2026 Sep 17;6(9):2187–205. doi: 10.1158/2767-9764.CRC-26-0305 (PMC13583505; doi:10.1158/2767-9764.CRC-26-0305)
Supplement: Supplementary file 13 [file crc-26-0305_figure_s9_suppsf9.pdf]

Supplementary Fig. 9

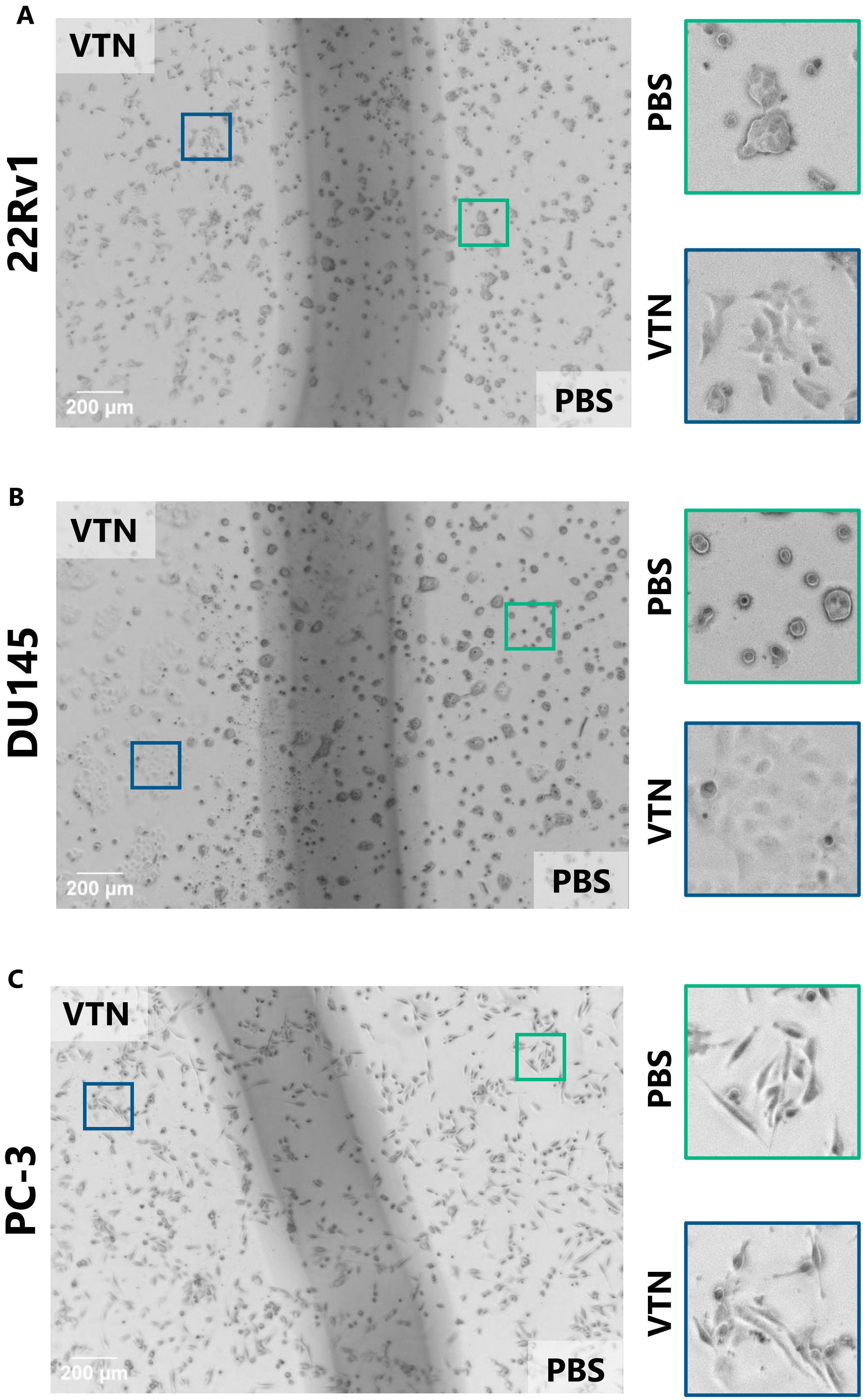

Figure S9. VTN foci promote differential spreading of 22Rv1 and DU145 but not PC-3.

(**A-C**) Gaskets were pressed firmly to the center of 10 cm plates and interior regions were coated with 80 µL spots of 5 µg/mL VTN in PBS as described. Space around the gasket was coated with PBS. After coating, excess PBS was aspirated, whole plates were washed with PBS, and cells were seeded in serum-free media. Images were taken 24 hours after seeding. The black marker down the center of the plate denotes placement of the gasket prior to coating and VTN or PBS-coated regions are indicated on either side of the mark.
